# Linking Individual Variability of Multi-modal Connectivity, Lifestyle, Psycho-Social Factors and Cognition in Older Adults

**DOI:** 10.14336/AD.2025.0428

**Published:** 2026-07-22

**Authors:** Mingxian Zhang, Susanne Moebus, Nico Dragano, Nora Bittner, Svenja Caspers

**Affiliations:** ^1^Institute of Neuroscience and Medicine (INM-1), Research Centre Juelich, Juelich, Germany.; ^2^Institute for Anatomy I, Medical Faculty & University Hospital Düsseldorf, Heinrich Heine University Düsseldorf, Düsseldorf, Germany.; ^3^Institute for Urban Public Health, University of Duisburg-Essen, Essen, Germany.; ^4^Institute of Medical Sociology, Medical Faculty & University Hospital Düsseldorf, Heinrich Heine University Düsseldorf, Düsseldorf, Germany.

**Keywords:** Multi-modal Connectivity, Lifestyle, Psycho-Social Factors, Cognition

## Abstract

Older adults exhibit greater heterogeneity than younger adults in behavior, cognition, and brain, which may be influenced by a range of factors, including lifestyle. While previous studies have assessed brain heterogeneity by evaluating the dissimilarity of individual brain connectivity, further empirical evidence is needed to understand the factors behind brain heterogeneity in older adults. Using data from the 1000BRAINS study (*N* = 461, aged 55-85 years), we analyzed the individual variability (IV) of the functional (IV_FC_) and structural (IV_SC_) connectivity across 421 brain regions. We aimed to explore the relationship between network-wise and region-wise brain connectivity IV (i.e., both IV_FC_ and IV_SC_), lifestyle, including psycho-social factors (e.g., self-reported smoking, physical activity, alcohol consumption, and social integration), and cognitive function via partial least squares correlation, stratifying analyses by age subgroups (55-64, 65-74, and ≥ 75 years), separately. Our results showed that higher connectivity IV was linked to lower social integration and/or higher smoking, and lower cognitive performance (e.g., episodic memory and executive control). For the network-wise analysis, we observed contributions from both IV_FC_ and IV_SC_ across eight networks, especially IV_SC_ in the salience and ventral attention networks. Region-wise, significant contributions came primarily from the connectivity IV of specific brain regions (e.g., inferior frontal gyrus, right anterior cingulate cortex). This result pattern varied by age group. Connectivity IV was positively correlated with smoking in the age 65-74 group and negatively correlated with alcohol consumption in the age ≥ 75 years group. Overall, IV_SC_ contributed more than IV_FC_ with age. These findings suggest that unhealthy lifestyle and social isolation might be associated with differences in neural resources, which may be linked to increased individual brain heterogeneity and, in turn, to lower cognitive performance in older adults, supporting the revised Scaffolding Theory of Aging and Cognition (STAC-r).

## INTRODUCTION

The heterogeneity of human cognitive function becomes more pronounced as we age [1]. While some older adults may successfully maintain their cognitive function into advanced age, others may experience significant cognitive decline or develop neurodegenerative diseases such as dementia, including Alzheimer's disease (AD) [2]. Research has shown that certain cognitive functions, notably episodic long-term memory [3] and processing speed [4], are sensitive to aging, whereas cognitive functions like language [5] and performance in tasks that involve knowledge [6, 7] are comparatively stable. Notably, even among healthy older adults with normal cognitive performance, heterogeneity persists across different cognitive domains. The heterogeneity can largely be attributed to differences in brain structure, functional properties (e.g., blood flow), or distinct structural and functional brain connectivity patterns [8, 9]. Understanding individual differences of brain structure and functional connectivity in the aging population may thus help predict the cognitive performance of older adults and develop personalized strategies for the prevention of neurodegenerative diseases in the future.

Therefore, research has increasingly focused on individual variability of the brain. Brain connectivity, which explains how different brain regions integrate and segregate information, has been suggested as a useful tool for studying such individual variability. It may even act as a unique ‘neural fingerprint’, enabling the identification of individuals and the prediction of specific traits (such as fluid intelligence) based on their unique patterns of brain connectivity [10-12]. Müller et al. (2013) proposed a simple and straightforward method for measuring individual differences in brain functional connectivity (FC) relative to the population, which reflects the spatial distribution of the individual differences in FC [9]. Their study and following observations from other researchers in recent years [13-18] have demonstrated that, generally, the association cortex shows higher individual variability of functional connectivity (IV_FC_), whereas sensorimotor and visual regions exhibit lower variance across individuals. These findings emphasize that functional connectivity provides a lens for exploring individual differences across various brain regions. These insights into the individual variability of connectivity (connectivity IV) have moreover been expanded by the investigation of diffusion MRI, which has further contributed to our understanding of the individual variability in structural properties of the brain. For example, Sun's study (2022) showed that individual variability in structural connectivity (SC) (IV_SC_) was generally lower relative to IV_FC_ in the population [17]. Specifically, IV_SC_ is lowest in sensorimotor networks, intermediate in association networks, and highest for para-limbic connections [19].

Age-related changes in functional and structural connectivity provide further information to understand the individual variability of the brain. Of particular interest is the variance of brain functional and structural connectivity patterns across different stages of late adulthood [20, 21]. For instance, participants between 60 and 69 years of age showed relatively consistent density and strength of FC. Participants between 70 and 79 years old showed lower strength of FC than other age groups, while the oldest group (≥ 80 years) exhibited slightly higher strength of FC than the 60-79 age groups [22]. Aging might be further associated with distinct connectivity profiles, where lower cognitive performance is associated with both lower SC and FC, while better cognitive performance is related to lower SC but higher FC [23]. In addition to the evidence that brain connectivity patterns vary across late adulthood, the IV_FC_, especially of the visual and subcortical networks, and IV_SC_, especially of the ventral attention network and default mode network (DMN), were also found to be positively correlated with age [15, 19]. Furthermore, when stratified into seven decade-based subgroups, the IV_FC_ was higher in the three older subgroups (aged 59-68, 69-78, and 79-88 years) than in four younger subgroups (aged 18-58 years), with the highest value observed in the 79-88 age group [15]. Together, these findings suggest that SC and FC can be used to capture individual differences in the brain, providing a methodological possibility for revealing the individual heterogeneity of the neural mechanisms of brain aging.

Given the relevance of connectivity IV (i.e., both IV_FC_ and IV_SC_), particularly in older age, the question arises: which factors might contribute to such pronounced variability? As modifiable behavioral factors, lifestyle is strongly associated with age-related differences in brain function, structure, and cognition [1]. The revised scaffolding theory of aging and cognition (STAC-r) suggests that positive lifestyle choices and lifetime experiences, including physical exercise, social engagement, and intellectual engagement, can enrich neural resources and enhance compensatory mechanisms, thereby helping to maintain cognitive function as the brain ages. In contrast, negative factors such as a sedentary lifestyle, smoking, and drinking may deplete neural resources, thereby weaken compensatory mechanisms and further impairing cognitive function [24-26]. For example, Steffener et al. (2016) found that, for every daily flight of stairs climbed, brain age decreases by 0.59 years, and for each year of schooling completed, brain age decreases by 0.95 years [27]. A study by Franz et al. (2021) found that unhealthy lifestyle factors (e.g., smoking, drinking alcohol, unhealthy diet, reduced physical and social activity) may accelerate brain aging in individuals with lower general cognitive performance, highlighting the importance of promoting healthy lifestyle habits [28]. Similarly, Bittner et al. (2021) found that for each packyear of smoking, brain age increased by 0.6 years, which might indicate accelerated structural brain aging, while physical activity was rather protective against brain aging in their cross-sectional study [29]. Exploring 25,378 UK Biobank participants, a study by Topiwala et al. (2022) suggested that higher alcohol consumption is associated with lower total gray matter volume (GMV), fractional anisotropy (FA) of widespread white-matter regions across the whole brain as well as higher functional connectivity within each network except the visual network in mid-to-late life [30]. A longitudinal study [31] highlighted the role of psycho-social factors, with better perceived social support being linked to larger total brain and gray matter volumes and slower reduction of total brain volume over time. Another study [32] using the UK Biobank imaging cohort further revealed that social support was most strongly associated with higher GMV in the salience network and the limbic network. Individuals with high social support showed higher FC between the salience, limbic, dorsal attention, and somatomotor networks, while FC between the DMN or frontoparietal control network and the visual network was lower. These studies underscored the nuanced relationship between lifestyle/psycho-social factors, cognition, and brain features, but did not take the individual variability of brain features among older adults as an influencing factor itself and have not explored the relationship between lifestyle/psycho-social factors, cognition, and brain heterogeneity during different stages of old age. Answering this open question will help us gain insight into the potential neural mechanisms of brain heterogeneity during aging, supplementing the empirical support for existing aging theories, such as STAC-r, and helping to develop neuroimaging markers that can predict cognition in future research.

Thus, our study aimed to explore the relationship between lifestyle/psycho-social factors, multiple cognitive domains, and brain connectivity IV in a large sample of 461 healthy older adults from the 1000BRAINS study, as well as its three age subgroups. We focused on five lifestyle and psycho-social factors, such as smoking, alcohol consumption, physical activity, and social integration. These factors were included based on prior studies linking them to brain structure, function, and cognition [24-32], as they represent both risk and protective domains. We chose to exclude factors that are multivariate themselves and would need intensive pre-evaluation, e.g., diet questionnaires. We assumed that the relationship between lifestyle/psycho-social factors, cognition, and connectivity IV may differ across age groups in late life.

## MATERIALS AND METHODS

### Participants

The participants for the current study were drawn from the 1000BRAINS study [33], originating from the 10-year follow-up of the epidemiological population-based Heinz Nixdorf Recall Study [34].

From the initial sample of 1000BRAINS (*n* = 1,314), 340 younger participants were excluded, leaving 974 participants aged from 55 to 87 to be considered for inclusion in the current study. Of these, we excluded participants with missing information on education level (measured by the International Standard Classification of Education (ISCED 1997, *n* = 4) [35], and potential cognitive impairment according to the dementia screening test (DemTect score ≤8, *n* = 26) [36]. Due to preprocessing failures of structural, diffusion, and/or functional image data, we excluded 245 participants (e.g., artifacts in MRI scans, problems during the normalization procedure, or AROMA-denoising) [23]. Finally, participants missing lifestyle and psycho-social measurements at more than two time points (*n* = 198), and/or those missing any of the 13 cognition measures of interest (*n* = 37) were excluded. The final study sample comprised 461 participants (age range 55-85 years old, female: *n* = 210, Table 1).

Written informed consent was obtained from all participants before inclusion in 1000BRAINS. The study protocol for 1000BRAINS was approved by the Ethics Committee of the University of Duisburg-Essen, Germany, and is in accordance with the declaration of Helsinki.

### Measures of lifestyle/psycho-social factors

Lifestyle/psycho-social data used here were retrieved from the database of the third examination of the Heinz Nixdorf Recall study (10-year follow-up), which commenced in June 2011 [33, 34]. We replaced missing values with the mean of the measurements from the first two time points. Here, we considered 5 domains of lifestyle and psycho-social factors: smoking, alcohol consumption, metabolic equivalent of the physical task (MET), flights of stairs climbed in daily life, and social integration.

For tobacco smoking, packyears of smoking were considered. It is calculated by multiplying the years of smoking with the self-reported number of smoked cigarettes per day (CPD) [29, 37, 38].

The alcohol consumption was assessed via a self-report interview asking about the average consumption of different beverages (e.g., beer, wine, spirits, and cocktails) within the last 4 weeks. The proportion of pure alcohol per beverage was then multiplied by the frequency of drinking and summed to get the total monthly consumption of pure alcohol (g/month).

The MET [39] is a measurement that shows the amount of energy people burn during exercise, sports, or any physical activity [40] relative to the resting metabolic rate. Participants were asked to report on their sports (e.g., running) and daily physical activities (e.g., gardening) carried out within the last month. Based on the compendium of physical activities, MET values were assigned to each of the activities reported by the participants, multiplied by the duration in hours (per month), and finally summed to obtain a total score for all activities.

Additionally, participants reported how many flights of stairs they usually need to climb daily. It is a valuable measure of cardiovascular fitness [41]. Climbing more flights of stairs may be related to better brain health [27].

Social integration, a psycho-social factor, was measured using an adapted version of the social integration index developed by Berkman et al. (2004) [42] (also see details in [43]). This index quantifies both structural aspects (e.g., marital status and number of social connections) and participatory aspects (e.g., engagement in social groups) of an individual's social life. It comprised three domains: first, “marital status”, where married or cohabitating participants had a score of 2, while single, never married, widowed, or divorced participants had a score of 0; second, close ties, which is determined by adding up the number of children, close relatives, and friends; and third, membership in organizations, based on the number of organizations in which an individual is active, participating at least once a month. The scores of all three domains were summed up into the social integration score.

### Neuropsychological performance tests

All participants underwent a comprehensive neuro-psychological assessment during the 1000BRAINS data collection, addressing a wide range of cognitive functions. In the current study, we included 13 different tests of cognitive function, such as cognitive status measured by the DemTect (indicating overall cognitive performance), episodic memory, long-term memory (LTM), verbal working memory (WM), as well as executive control (see details in Supplementary Table 1; also see [33, 44]).

### Magnetic resonance imaging (MRI)

Image acquisition: Magnetic resonance imaging (MRI) was carried out on a 3T Siemens Tim-TRIO MR scanner with a 32-channel head coil (Erlangen, Germany) during the 1000BRAINS data collection, on the same day as the neuropsychological examination. The study protocol is described in detail in Caspers et al. (2014) [33]. For surface reconstruction and spatial registration, high-resolution T1-weighted imaging was used: 176 slices, slice thickness 1 mm, repetition time (TR) = 2250 ms, echo time (TE) = 3.03 ms, field of view (FoV) = 256 × 256 mm^2^, flip angle = 9, voxel resolution 1 × 1 × 1 mm^3^. For structural connectivity analyses, high-angular resolution diffusion imaging (HARDI) data was used: (1) 120 directions subset; echo planar imaging (EPI) sequence, TR = 8 s, TE = 112 ms, 13 *b*0-images (interleaved), 120 images with *b* = 2700 s/mm^2^, voxel resolution = 2.4 × 2.4 × 2.4 mm^3^; (2) 60 directions subset (with directions sampled from those of 120 direction dataset); EPI, TR = 6.3 s, TE = 81 ms, 7 *b*0-images (interleaved), 60 images with *b* = 1000 s/mm^2^, voxel resolution = 2.4 × 2.4 × 2.4 mm^3^. For functional connectivity analyses, the resting-state functional MRI (rsfMRI) data were acquired as a blood-oxygen-level-dependent (BOLD) gradient-EPI sequence with 36 transversal slices (slice thickness 3.1 mm, TR = 2200 ms, TE = 30 ms, FoV = 200 × 200 mm^2^, voxel resolution 3.1 × 3.1 × 3.1 mm^3^), lasting ~11 min and producing 300 volumes. During scanning, participants were instructed to keep their eyes closed and remain relaxed, but not to fall asleep.

Image preprocessing: Preprocessed data were derived from a previous study [23]. Details of the preprocessing process, visual image quality checks, and exclusion criteria for MR images can be seen in the supplementary material and [23].

In short, tissue probability maps (TPMs) for gray matter (GM), white matter (WM), and cerebrospinal fluid (CSF) were created from T1-weighted images using the Computational Anatomy Toolbox (CAT12; https://neuro-jena.github.io/cat/) [45]. Then, T1-weighted images were automatically bias-field corrected, aligned to the MNI152 template, and resampled to 1.25 mm isotropic voxel size using the FSL toolbox (FMRIB Software Library: www.fmrib.ox.ac.uk/fsl) [46].

For diffusion MRI (dMRI) data, eddy current and head motion artifact correction were performed first. To co-register dMRI to T1-weighted images, the first *b*0 images from each dMRI data with *b*1000 and *b*2700 were extracted and rigidly aligned to T1-weighted images, separately. To address susceptibility artifacts, anisotropic power maps (APM) [47] were computed from dMRI data with *b*2700 and were non-linearly registered to T1-weighted images using ANTs (https://stnava.github.io/ANTs/). This resulted in a nonlinear transformation matrix, which was then used to transform the TPMs into diffusion space. Finally, the dMRI with *b*1000 and *b*2700 were merged into one single file and corrected for different echo times. For tractography, the constrained spherical deconvolution (CSD) local model was computed using multi-tissue CSD of multi-shell data [48] from the MRtrix software package (version 0.3.15) [49]. Then, using the probabilistic iFOD2 algorithm, ten million streamlines were computed with dynamic seeding in the gray-white matter interface for every participant with a maximal length of 250 mm and a cut-off value of 0.06.

For rsfMRI data, the first four volumes of each participant’s rsfMRI data were discarded. Then, residual volumes were corrected for head movement using rigid-body registration in a two-pass procedure. After that, we used ICA-AROMA to identify and remove motion-related components from the data. Combined AROMA with global signal regression to minimize motion-related effects on functional connectivity. All rsfMRI data were band-pass filtered (0.01-0.1 Hz) and registered to the MNI152 space template using the unified segmentation approach [50]. All these steps were applied using the FSL toolbox.

### Brain connectivity

To analyze FC and SC data, we used a combined atlas with 421 brain regions in total, comprising 400 cortical parcels of the parcellation by Schaefer et al. (2018) [51], 14 sub-cortical regions [52], as well as 7 cerebellar regions [53]. For the sub-cortical regions, we used the file “Atlas_ROIs.2.nii.gz” derived from FreeSurfer (version 5.3.0). We excluded five regions of no interest for the current study (i.e., brainstem and cerebellar regions), keeping the remaining 14 sub-cortical regions. For the cerebellar regions, we used an integrative functional atlas of the human cerebellum presented by Buckner et al. (2011) [53]. These cortical and cerebellar regions have been pre-assigned to Yeo’s seven networks [51, 53]. Sub-cortical brain regions were then combined into one subcortical network according to previous studies [54, 55].

Regarding FC, the average time series of all voxels within 421 brain regions were extracted from preprocessed rsfMRI data using fslmeants [56]. Then, Pearson’s correlation coefficient of the time series between any pair of brain regions was computed to construct a 421 × 421 FC matrix. According to previous studies [57, 58], to minimize the number of edges resulting from spurious, noise-related correlations, we excluded statistically non-significant correlation coefficients in an additional step using a permutation test (iteration *n* = 1,000 times, non-significant edges at *p* ≥ 0.05 were set to zero). Finally, we performed Fisher’s *r*-to-*z* transformation to normalize the correlation coefficient of the individual FC.

Regarding SC, the parcellation template with 421 regions was first registered to the individual diffusion space. The predefined 400 Schaefer’s parcellation template primarily covers cortical gray matter. To ensure that the parcellation scheme adequately covers the gray-white matter interface and can serve as seeding points for probabilistic streamline tractography, we dilated the template boundary by 5 voxels [23]. Furthermore, to increase the biological accuracy of SC, we converted streamline counts between each pair of nodes into weighting factors using a cross-sectional area multiplier (SIFT-2) [59]. Finally, the derived 421 × 421 matrix was log10 transformed.

### Measuring the connectivity IV

According to Mueller et al. [9], individual variability of connectivity describes how much the connectivity matrix of a participant deviates from those of all other participants in a group. It is calculated as the reverse of the similarity in brain connection patterns between one individual and other members within the group, providing a measure of dissimilarity reflecting the variability in the group (see also Supplementary Fig. 1 for details). The code for calculating connectivity IV (i.e., both IV_FC_ and IV_SC_) was adapted from Sun et al. (2022) [17]. Specifically, taking FC as an example, we took the following two steps to calculate connectivity IV for each participant:

1. Individual similarity of FC (IS_FC_) was calculated for each participant *m,* at a given brain region *i* (*i* = 1, 2, … 421), as the average correlation value between *m*’s FC and the FC of all other participants (*N-1*):
$${\mathrm{IS}}_{\mathrm{FC}i}\mathrm{\_m}=\frac{\sum \mathrm{corr}({\mathrm{FC}}_{i}\mathrm{\_m},{\mathrm{FC}}_{i}\mathrm{\_n})}{N-1}$$
  Where *m, n* = 1, 2, … 461 (participants of the sample), and *m* ≠ *n*. *N* is the total number of participants. ${\mathrm{FC}}_{i}\_m/n$ is the FC of region *i* to all other regions of participant *m*/*n.*
2. Individual variability of FC (IV_FC_) for participant *m* at a given region *i* (*i* = 1, 2, … 421) was then derived by subtracting their IS_FC_ from 1:

IV_FC_*_i__m_=_* 1 - IS_FC_*_i__m*

This transformation converts similarity (i.e., IS_FC_, ranging between -1 and 1) to dissimilarity (i.e., IV_FC_, ranging between 0 and 2), where higher IV_FC_ values indicate greater distance/dissimilarity of the FC of region *i* for this individual from the group-average FC pattern of that region *i*.

The individual variability of SC (IV_SC_*_i__m*) was calculated similarly. Finally, we obtained 461×421 IV_FC_ and IV_SC_ matrices that will be used in the main analysis.

To gain a comprehensive understanding of connectivity IV, although participant-level individual variability of connectivity was the focus of our study (mentioned above), additional supplemental analyses for group-level individual variability of connectivity were performed and can be found in the supplementary material.

### Partial least squares correlation

**Figure 1.**
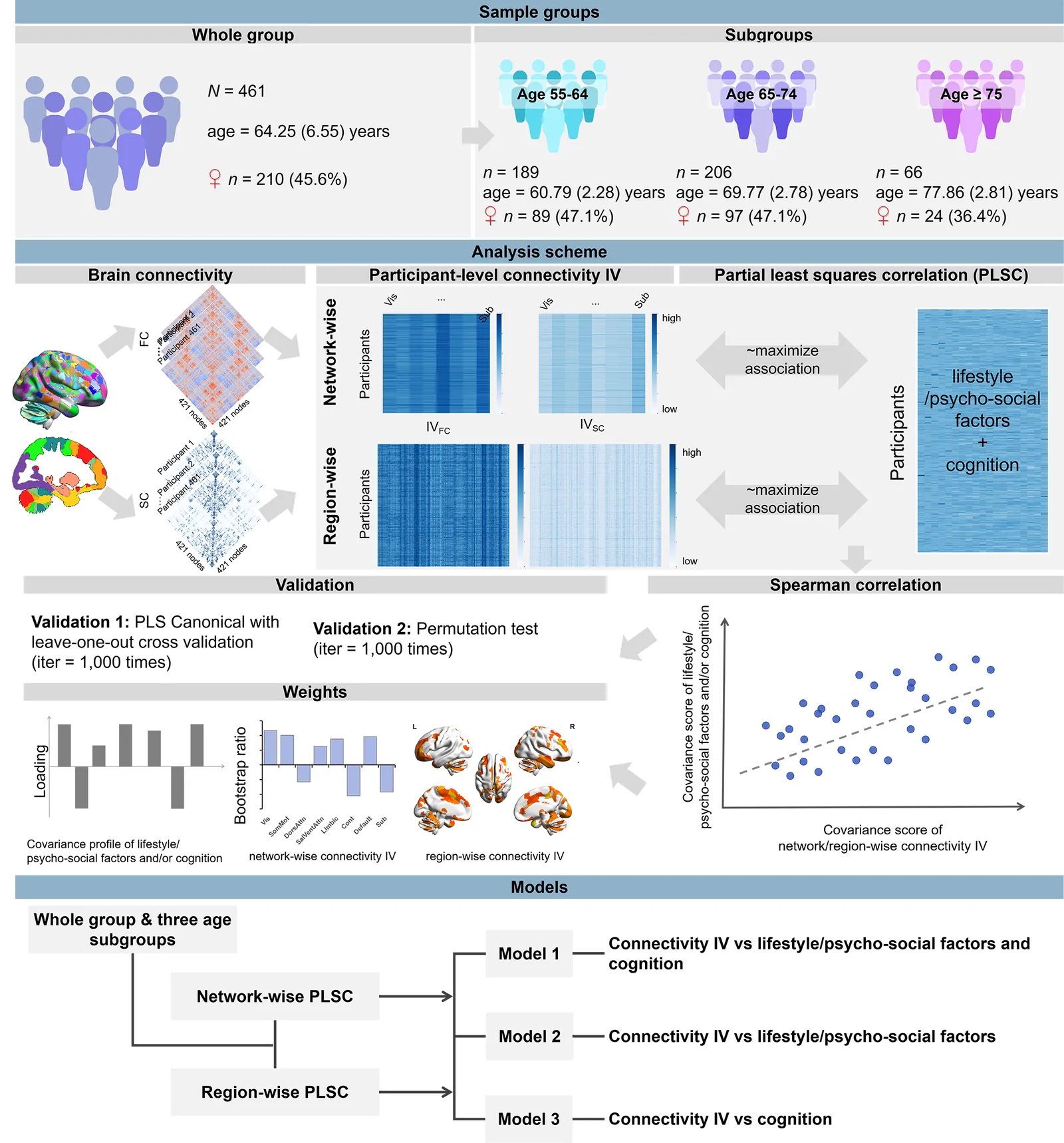
**Overview of partial least squares correlation (PLSC) framework and analysis design**. Top panel: Full sample and its division into three age groups. Middle panel: Analysis scheme. Functional and structural connectivity were computed from 421 regions (400 cortical, 14 subcortical, 7 cerebellar). Inter-individual variability (IV) of connectivity was calculated at both network and region levels. Network-wise connectivity IV was averaged across Yeo’s 7 cortical and one subcortical networks. Region-wise connectivity IV was retained at the parcel level. These brain measures, along with lifestyle/psycho-social and/or cognitive factors, were input into PLSC to identify latent variables (LVs) capturing maximal brain-behavior & cognition covariance. We focused on the first LV. Associations were assessed with Spearman correlation and validated by two methods. Results are displayed as loadings of lifestyle/psycho-social and cognitive factors, and a bootstrap ratio plot for connectivity IV (bar charts for network-wise connectivity IV; brain maps for region-wise connectivity IV). Bottom panel: Three models with different input features were applied to the whole group and age subgroups, separately for network- and region-wise analyses. Abbreviations: Vis, visual network; Sub, subcortical network

To systematically investigate the relationship between brain connectivity IV, lifestyle/psycho-social factors, and cognition, we conducted a partial least squares correlation (PLSC) analysis using the pypyls package (https://github.com/netneurolab/pypyls). We implemented three models (lifestyle/psycho-social factors only, cognition only, and combined) at both the network and region level across the whole group and three age subgroups (see Fig. 1). This multi-level, multi-model framework enables us to evaluate the robustness and specificity of associations, as well as to examine age-dependent patterns. PLSC is a multivariate analysis method and is favored for its robustness in handling multicollinearity and capturing nonlinear relationships, allowing for a comprehensive analysis across multiple variables [60, 61]. It is increasingly being used to explore the relationship between the brain and behavior [62-66]. It determines the relationship between two sets of variables, *X* and *Y*, by yielding latent variables that maximize the covariance between linear combinations of the original variables [67]. For example, in our case, each LV would represent a pattern of covariation between brain connectivity, lifestyle/psycho-social factors, and cognition, summarizing the most prominent linear relationships within the data. This approach enables the identification of brain-behavior & cognition associations that might not be evident through univariate analyses. The code for conducting the PLSC analysis was modified based on the work of Petersen et al. (2024) [66].

#### Network-wise analysis

To perform a network-wise analysis, we averaged the IV_FC_ and IV_SC_ values of 421 brain regions across eight networks separately. Then we built three models to study the inter-relation between connectivity IV, lifestyle/psycho-social factors, and cognition. In Model 1, the *X* matrix (461 participants × 16) consisted of the two IV_FC_ and IV_SC_ matrices, with a size of 461 participants × 8 networks each. The selected five lifestyle/psycho-social variables (packyears of smoking, alcohol consumption, MET, stair climbing, and social integration) and 13 cognitive variables were treated as the *Y* matrix (461 participants × 18 lifestyle/psycho-social, cognitive variables). Both *X* and *Y* matrices served as features in the PLSC analysis.

Before conducting the PLSC analysis, we normalized all the features and regressed out age, sex, and education to control confounding effects. The PLSC began by computing a correlation matrix between the brain data (*X* matrix) and the behavior and cognitive data (*Y* matrix). It then applied singular value decomposition (SVD, see also the supplementary material) to extract components known as latent variables (LVs). Each LV consists of a pair of weighted patterns, one for the *X* matrix and one for the *Y* matrix, that jointly capture a maximally covarying relationship between the two matrices. In other words, a latent variable summarizes the association between two dimensions (represented by the matrices) by identifying specific brain networks that covary with specific lifestyle/psycho-social variables, which in turn also covary with specific cognitive variables.

For each participant, covariance scores were calculated separately for the *X* and *Y* matrices by projecting their individual values onto the corresponding singular vectors. These scores reflect how strongly each individual expresses the brain-behavior & cognition pattern represented by the LV. To assess the contribution of each variable in the *Y* matrix to the identified LV, we performed bootstrap resampling (*n* = 5,000). The significance of each feature's contribution to the covariance pattern in the *Y* matrix was evaluated using singular vector weights and their confidence intervals (95% CI), which can be interpreted as similar to the weights of factor loadings in a principal component analysis. A variable was considered to make a significant contribution to the covariance pattern if its 95% CI did not include zero, indicating a stable and non-random association with the latent variable. Subsequently, to determine the contribution of network-wise IV_FC_ and IV_SC_ to the covariance patterns, bootstrap ratios were calculated, which are equal to z-scores derived from the normal distribution bootstrap method (see the supplementary material). Brain networks were considered significant contributors when the bootstrap ratio was < -1.96 or > 1.96. Together, the significantly contributing networks (covariance pattern 1) and the significantly cognitive and lifestyle variables (covariance pattern 2) reflect the latent variable and thus capture the maximized association between all of these variable dimensions.

Finally, we calculated a Spearman correlation between the covariance scores from the *X* and *Y* matrices across participants. A strong correlation (*r_s_*) indicates the consistency between the brain and behavior & cognition expressions of the latent variable. To validate the robustness of the correlation, we performed the Partial Least Squares Canonical (PLSCanonical) analysis using Leave-One-Out cross-validation (LOOCV) with 1,000 iterations [66]. For each iteration, we computed correlation coefficients and corresponding *p*-values with statistical significance defined as *p* < 0.05. To correct for multiple comparisons, we applied a false discovery rate (FDR) correction with a threshold of *q* < 0.05 [68]. Additionally, we performed a more conservative permutation test (iterated 1,000 times). It determined if the correlation coefficients obtained from PLSC and Spearman correlation were higher than the chance level. For the permutation test, a result was considered significant if the permutation-derived *p* < 0.05. Both methods were used to assess the robustness of the results, i.e., if the significance threshold was met in either the LOOCV method or the permutation test, the corresponding correlation was reported as significant.

Further, to assess the consistency of the results among different age subgroups, we conducted PLSC analysis not only in the whole group (*N* = 461), but also separately in three age subgroups (55-64 years, 65-74 years, and ≥ 75 years). In addition, to clarify the independent effects of lifestyle/psycho-social factors and cognitive variables, we also built Model 2 (lifestyle/psycho-social variables only in the *X* matrix) and Model 3 (cognitive variables only in the *X* matrix) and performed the same analysis.

#### Region-wise analysis

Considering that the network-wise analysis may obscure information about specific brain regions, we conducted the same analysis at the regional level to determine the contribution of particular brain regions to the relationship between connectivity IV, lifestyle/psycho-social factors, and cognition. In this case, we treated the IV_FC_ and IV_SC_ of 421 brain regions as the *X* matrix (461×842), while keeping the other inputs, parameters, and steps (i.e., building Models 1, 2, and 3 and separating age subgroups) consistent with the network-wise analysis. The bootstrap ratio was calculated to determine the contribution of brain regions to the model. In both network-wise and region-wise PLSC analysis, we focused only on the first latent variable in each model as it explained more variation across the included variables.

Involved regions were anatomically assigned using the JulichBrain Atlas [69].

#### Sensitivity analysis

The network-wise and region-wise PLSC analyses for Model 1 in the whole group were rerun after removing outliers on any lifestyle/psycho-social factors and cognition variables. An outlier was defined as any value above the mean value plus or minus three standard deviations. After removing the outliers, the sample consisted of 403 remaining participants for the sensitivity analysis.

### Statistics

We first examined the differences in demographic and neuropsychological variables between the groups. For normally distributed variables, we used Analysis of Variance (ANOVA) to compare differences among the three subgroups. For sex, between-group differences were tested by the chi-squared tests. A non-parametric Kruskal-Wallis test was used to measure non-normally distributed variables. For completeness, we also listed the characteristics of group-level connectivity IV and performed ANOVA to compare differences among the three age subgroups separately in different networks (results shown in Supplementary Table 3). These statistical analyses were carried out using IBM SPSS Statistics 29.0. The PLSC, Spearman correlation analysis, validation analysis, and plotting were performed in Python (version: 3.10.9), including packages: pandas (version: 1.5.3), NumPy (version: 1.23.5), pyls (version: 0.18), pingouin (version: 0.5.3), seaborn (version: 0.12.2), scipy (version: 1.10.0), Scikit-learn (version: 1.2.1), matplotlib (version: 3.7.0).

**Table 1:** Basic information about participants.

	Mean (SD)				Statistic	
	Whole group (*N* = 461)	Age 55-64 (*n* = 189)	Age 65-74 (*n* = 206)	Age ≥ 75 (*n* = 66)	*ᵡ^2^*/*F*-value	*p*-value
**Demographic variables**						
**Age (years)**	67.25 (6.55)	60.79 (2.28)	69.77 (2.78)	77.86 (2.81)	/	/
**Females (*n*, %) ^a^**	210, 45.6%	89, 47.1%	97, 47.1%	24, 36.4%	2.623	0.269
**Education (ISCED)^b^**	6.39 (1.95)	6.54 (1.91)	6.46 (1.95)	5.79 (1.98)	8.428	**0.015**
**Lifestyle/psycho-social factors**						
**Packyears (smoking in pack years) ^b^**	12.85 (20.99)	12.45 (21.29)	14.01 (21.23)	10.41 (19.37)	1.817	0.403
**Alcohol (in g/week) ^b^**	10.36 (17.72)	11.68 (18.27)	9.16 (17.57)	10.32 (16.58)	4.248	0.120
**MET (per week) ^b^**	46.19 (51.84)	44.77 (60.05)	48.46 (45.71)	43.14 (44.39)	6.064	**0.048**
**Stairs (number per day) ^b^**	6.75 (9.22)	8.04 (11.25)	6.62 (8.02)	3.48 (4.13)	14.879	**<0.001**
**Social integration (as social integration index) ^b^**	12.74 (6.38)	12.59 (5.81)	12.98 (6.66)	12.42 (7.10)	2.212	0.331
**Cognitive variables**						
**Cognitive status (sum) ^b^**	14.86 (2.34)	15.24 (2.36)	14.88 (2.26)	13.73 (2.24)	17.411	**0.001**

*Note*: The mean value and standard deviation are resented as mean (*SD*). Statistical analysis performed among three age subgroups.

a Chi-Square Tests

b Independent-Samples Kruskal-Wallis Test

Abbreviations: ISCED, International Standard Classification of Education; Packyears, packyears of smoking; Alcohol, alcohol consumption; MET, metabolic equivalent of the task; Stairs, flights of stairs climbed

**Figure 2.**
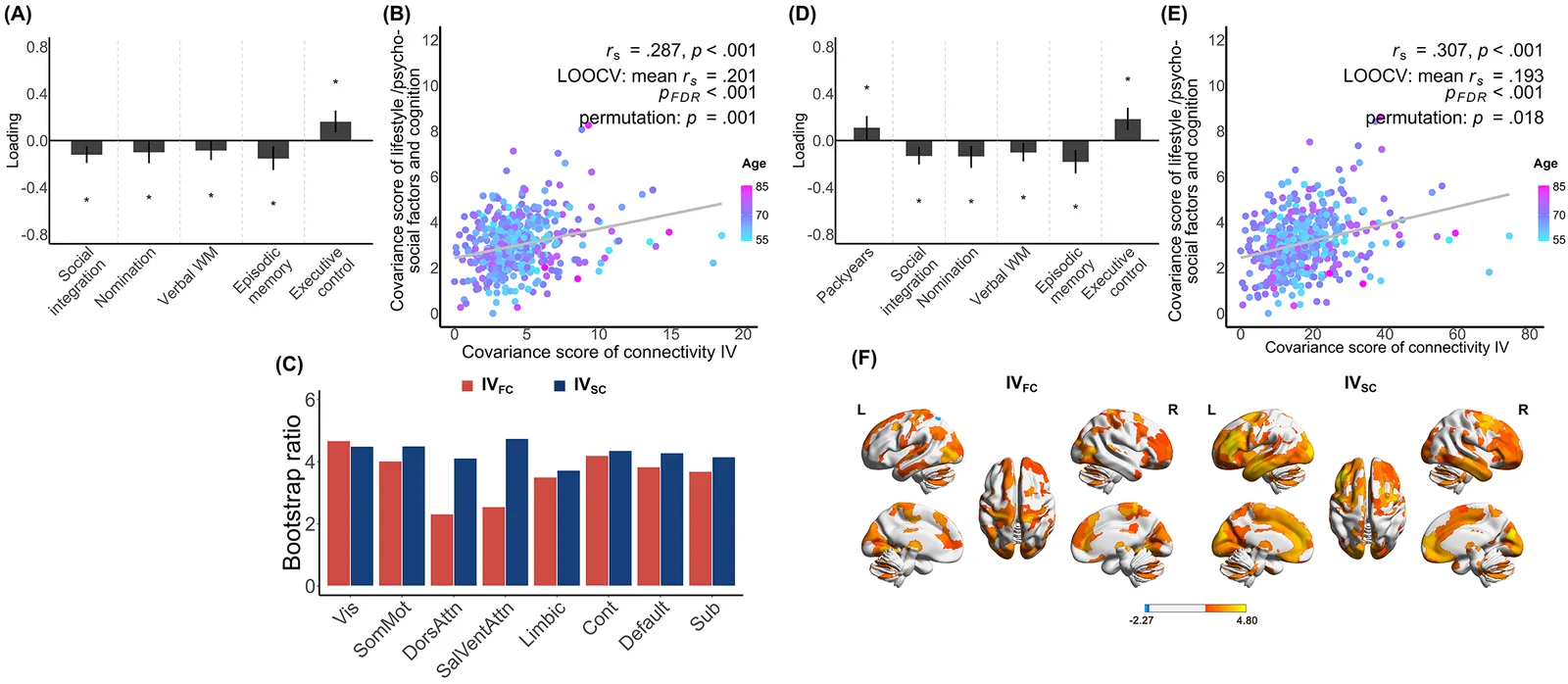
**Model 1 of network-wise (A-C) and region-wise (D-F) partial least squares correlation (PLSC) analysis in the whole group (N = 461)**. The first latent variable was used to investigate the latent relationship. (A, D) Loadings of lifestyle/psycho-social and cognitive variables with 95% confidence intervals (bootstrap resampling). Only significant variables are shown (*). (B, E) Scatterplots of covariance scores: individual variability (IV) of connectivity (X-axis) vs. lifestyle/psycho-social and cognitive scores (Y-axis), color-coded by age (55-85 years, light blue to pink). (**C**) Bootstrap ratio bar chart for network-wise IV of functional (IV_FC_, red) and structural connectivity (IV_SC_, blue). ‘ns’ marks non-significant results (-1.96 < ratio < 1.96). (**F**) Bootstrap ratio brain maps of region-wise IV_FC_ (left) and IV_SC_ (right). Warm (red-yellow) and cool (blue) colors represent positive and negative contributions. Only significant regions (|ratio| > 1.96) are shown. Confounders (age, sex, education) were regressed out before PLSC. Abbreviations: rs, Spearman correlation coefficient; Packyears, packyears of smoking; WM, working memory; Vis, visual network; SomMot, somatomotor network; DorsAttn, dorsal attention network; SalVentAttn, salience/ventral attention network; Limbic, limbic network; Cont, frontoparietal control network; Default, default mode network; Sub, subcortical network.

## RESULTS

### Demographic and neuropsychological measurements

First, we analyzed differences between the whole sample and the age subgroups in demographic, lifestyle/psycho-social, and cognitive variables. Table 1 lists basic information and descriptive statistics of the whole group (*N* = 461, mean age = 67.25±6.55 years old, mean education level = 6.39±1.95) and its three subgroups based on age. The results showed that, given the increasing age, expected between-group differences in education level, metabolic equivalent of the physical task (MET), flights of stairs climbed, and 12 cognitive domains (except for phonemic fluency), e.g., the oldest age group showed the lowest performance in cognitive status (details are listed in Supplementary Table 2). Therefore, these results are in line with our hypotheses, and age subgroups were analyzed separately and not directly compared to each other.

### PLSC analysis for the whole group

#### Factors related to network-wise connectivity IV

Network-wise Model 1: combined model. Model 1 examined the relationship between network-wise connectivity IV (i.e., both IV_FC_ and IV_SC_), lifestyle/psycho-social factors, and cognition (see details in Fig. 2). A significant latent variable (LV) explained 73.89% of the shared variance among all variables (*p* < 0.001; see Supplementary Table 4 for details of LVs of Models 1-3, including the proportion of explained variance and *p*-values).

Covariance scores of the connectivity IV positively correlated with those of lifestyle/psycho-social factors and cognition (*r_s_* = 0.287, *p* < 0.001), indicating that higher connectivity IV was associated with a higher covariance score of lifestyle/psycho-social factors and cognition. This relationship was robust across LOOCV (mean *r_s_* = 0.201, *p*_FDR_ < 0.001) and permutation testing (*p* = 0.001; panel B of Fig. 2).

The contribution of each specific variable to the shared pattern of lifestyle/psycho-social factors and cognitive functions is represented by weights [lower CI; upper CI]. Social integration (-0.12 [-0.19, -0.05]) had a significantly negative relationship with connectivity IV, suggesting that individuals with lower social integration exhibited higher network-wise connectivity IV. Meanwhile, lower performance in several domains was linked to higher connectivity IV: nomination (-0.10 [-0.19, -0.01]), verbal working memory (WM) (-0.09 [-0.17, -1.33×10^-3^]), episodic memory (-0.15 [-0.25, -0.05]), and executive control (0.16 [0.07, 0.26]; measured in time, a higher value reflects lower performance) (Fig. 2A; also see Table 2 for variable loadings). Connectivity IV, contributed positively to the LV, specifically driven by the IV_SC_ of the salience/ventral attention (SalVentAttn) network (bootstrap ratio = 4.745; see Figure 2C and Table 3 for network contributions). Overall, the identified pattern (i.e., LV) summarized a significant relationship between higher connectivity IV, lower social integration, and concurrent lower cognitive performances in the described domains.

**Table 2:** Loading of the lifestyle/psycho-social factors, and/or cognition covariance pattern of the network-wise partial least squares correlation (PLSC) analysis in the whole group.

Variables	Model 1		Model 2		Model 3	
	Loading	CI (lower, upper)	Loading	CI (lower, upper)	Loading	CI (lower, upper)
Packyears	0.09	-0.01, 0.18	0.10	-0.01, 0.19	/	/
Alcohol	0.07	-0.01, 0.15	**0.07**	**4.65×10^-5^, 0.16**	/	/
MET	-0.03	-0.12, 0.07	-0.03	-0.11, 0.07	/	/
Stairs	0.01	-0.06, 0.10	0.02	-0.06, 0.10	/	/
Social integration	**-0.12**	**-0.19, -0.05**	**-0.12**	**-0.19, -0.05**	/	/
Cognitive status	-0.01	-0.11,0.09	/	/	-0.01	-0.11,0.08
Language comprehension	-0.07	-0.18,0.03	/	/	-0.07	-0.18,0.03
Nomination	**-0.10**	**-0.19, -0.01**	/	/	**-0.10**	**-0.19, -0.02**
Figural memory	-0.06	-0.15,0.03	/	/	-0.06	-0.14, 0.02
Verbal WM	**-0.09**	**-0.17, -1.33×10^-3^**	/	/	**-0.08**	**-0.17, -9.20×10^-4^**
Phonemic fluency	0.00	-0.09, 0.09	/	/	0.00	-0.09, 0.09
Phonemic switch	0.07	-0.03, 0.18	/	/	0.07	-0.03, 0.18
Semantic fluency	-0.02	-0.11, 0.07	/	/	-0.02	-0.11, 0.07
Semantic switch	0.03	-0.05, 0.12	/	/	0.03	-0.05, 0.11
Visuospatial WM	-0.02	-0.10, 0.07	/	/	-0.01	-0.10, 0.08
Episodic memory	**-0.15**	**-0.25, -0.05**	/	/	**-0.15**	**-0.26, -0.05**
Long-term memory	-0.04	-0.14, 0.05	/	/	-0.04	-0.14, 0.06
Executive control	**0.16**	**0.07, 0.26**	/	/	**0.16**	**0.07, 0.26**

*Note*: A significant loading is marked as bold. Abbreviations: CI, 95% confidence intervals; Packyears, packyears of smoking; Alcohol, alcohol consumption; MET, metabolic equivalent of the task; Stairs, flights of stairs climbed; WM, working memory

Network-wise Model 2: lifestyle/psycho-social factors only. In Model 2, a significant LV (explaining 76.46% of variance, *p* = 0.002; see Supplementary Table 4 for full model details) linked higher alcohol consumption (0.07 [4.65×10^-5^, 0.16]) and lower social integration (-0.12 [-0.19, -0.05]) to higher IV_FC_ of 6 networks, especially of the frontoparietal control (Cont) network, and higher IV_SC_ of 7 networks, especially of the SalVentAttn, partially consistent with Model 1 findings (*r_s_* = .159, *p* < 0.001; LOOCV: mean *r_s_* = .102, *p_FDR_* = 0.028; permutation: *p* = 0.099; see details in Supplementary Fig. 2, Tables 2-3).

Network-wise Model 3: cognition only. Similar to Model 1, Model 3 identified a significant LV (explaining 74.35% of variance, *p* < 0.001; see Supplementary Table 4 for detailed results), linking higher network-wise connectivity IV to lower performance in nomination, verbal WM, episodic memory, and executive control (*r_s_* = 0.240, *p* < 0.001; LOOCV: mean *r_s_* = .164, *p_FDR_* < 0.001; permutation: *p* = 0.002; see details in Supplementary Fig. 2 and Tables 2-3). The pattern of this LV is almost in line with the contribution of brain connectivity IV patterns in Model 1, except for the IV_FC_ of the dorsal attention (DorsAttn) and SalVentAttn networks.

#### Factors related to region-wise connectivity IV

Region-wise Model 1: combined model. We next conducted region-wise analyses to explore how connectivity IV of specific brain regions relates to lifestyle/psycho-social factors and cognition (see details in Fig. 2). In Model 1, one significant LV was identified in the whole group (explaining 40.06% of variance, *p* < 0.001; see Supplementary Table 7 for details of LVs of Models 1-3). A moderate positive correlation emerged between the covariance score of region-wise connectivity IV and those of lifestyle/psycho-social factors and cognition (*r_s_* = 0.307, *p* < 0.001, LOOCV: mean *r_s_* = 0.193, *p_FDR_* = <0.001; permutation *p* = 0.018; Fig. 2E). This association was primarily driven by higher packyears of smoking (0.11 [0.01, 0.21]) and lower social integration (-0.13 [-0.20, -0.06]), alongside lower performance in executive control (0.18 [0.09, 0.28]), episodic memory (-0.18 [-0.28, -0.08]), nomination (-0.14 [-0.23, -0.05]), and verbal WM (-0.10 [-0.18, -0.02]) (Fig. 2D; see also Table 4). For region-wise connectivity IV, positive contributions (i.e. higher connectivity IV of multiple regions) were found in: (i) IV_FC_, mainly regions within the visual network, particularly the left fusiform gyrus (area FG3 [70]), left visual cortex (area hOc2 (V2, area 18 [71]), and the left subiculum of the hippocampal complex [72, 73]; (ii) IV_SC_, mainly regions of the frontoparietal control, default mode, and visual networks, in particular the bilateral anterior cingulate cortex (ACC, area 33 [74]), left inferior frontal gyrus (area 45), left frontal operculum (area OP9 [69]), right visual cortex (area hOc2), and right cuneus (area hOc3d [75]) (Fig. 2F).

**Table 3:** Bootstrap ratio of network-wise partial least squares correlation (PLSC) analysis in the whole group.

	IV_FC_			IV_SC_		
	Model 1	Model 2	Model 3	Model 1	Model 2	Model 3
Vis	**4.674**	**2.444**	**4.207**	**4.496**	**2.756**	**3.665**
SomMot	**4.019**	**2.330**	**3.334**	**4.503**	**2.321**	**3.759**
DorsAttn	**2.316**	1.612	1.651	**4.116**	**2.282**	**3.546**
SalVentAttn	**2.551**	**2.012**	1.804	**4.745**	**3.245**	**3.680**
Limbic	**3.506**	1.489	**3.275**	**3.726**	1.927	**3.340**
Cont	**4.199**	**3.501**	**3.057**	**4.361**	**3.016**	**3.586**
Default	**3.832**	**2.612**	**3.023**	**4.285**	**3.348**	**3.303**
Sub	**3.685**	**2.175**	**3.160**	**4.155**	**3.029**	**3.251**

*Note*: A significant bootstrap ratio is marked as bold. Abbreviations: IV_FC/SC_, individual variability of functional/ structural connectivity; Vis, visual network; SomMot, somatomotor network; DorsAttn, dorsal attention network; SalVentAttn, salience/ventral attention network; Limbic, limbic network; Cont, frontoparietal control network; Default, default mode network; Sub, subcortical network.

Together, these results indicated that higher connectivity IV of the significantly contributing regions was associated with higher packyears of smoking, less social integration, and lower cognitive performance. Conversely, IV_FC_ of one lateral occipital region showed a negative association, suggesting an inverse relationship pattern.

Region-wise Model 2: lifestyle/psycho-social factors only. In Model 2, higher packyears of smoking, alcohol consumption, and lower social integration were linked to higher connectivity IV of brain regions within the somatomotor (SomMot) and frontoparietal control networks (*r_s_* = .214, *p* < 0.001; LOOCV: mean *r_s_* = 0.098, *p_FDR_* = 0.035; permutation *p* = .385; see details Supplementary Fig. 3). The loadings of lifestyle/psycho-social variables contributing to this association are detailed in Table 4. Fewer clusters emerged than in Model 1. Specifically, key contributing regions included the right inferior frontal gyrus (area 45 [76, 77]), medial prefrontal cortex, middle temporal gyrus, ACC (area 33), left precentral gyrus (area 4a [78]), right postcentral gyrus (area 1 [79, 80]), superior parietal lobule (left area 5M [81, 82]; right area 5L), and precuneus.

Region-wise Model 3: cognition only. In Model 3, lower performance on five cognitive tests was associated with higher region-wise connectivity IV across widespread regions, largely overlapping with Model 1 (*r_s_* = 0.269, *p* < 0.001; LOOCV: mean *r_s_* = 0.155, *p_FDR_* = 0.001; permutation *p* = .134; see details in Supplementary Fig. 3 and Table 4).

**Figure 3.**
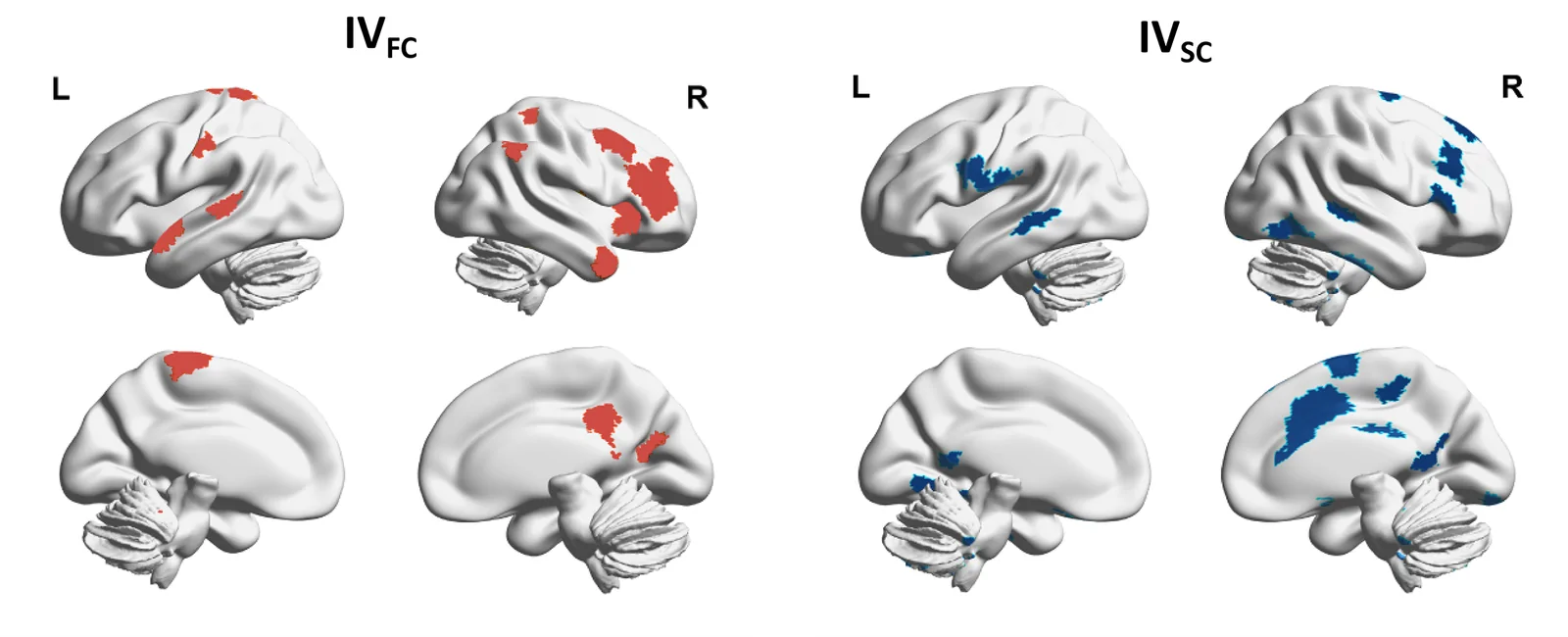
**The overlapping regions of significant contributions from Model 1 (connectivity IV-lifestyle/psycho-social factors-cognition relationship) and Model 2 (connectivity IV-lifestyle/psycho-social factors relationship) in the whole group**. The red color signifies IV of functional connectivity (IV_FC_), and the blue color represents IV of structural connectivity (IV_SC_). Abbreviations: IV, individual variability; L, left; R, right.

We have overlaid the bootstrap ratio brain maps of Models 1 and 2 to identify the regions that contribute to the connectivity IV-lifestyle/psycho-social factors relationship, although these brain regions may also be related to specific cognitive functions (Fig. 3). It illustrates that different brain networks involved in IV_FC_ compared to IV_SC_: (i) IV_FC_, regions mainly belonged to the SomMot, Cont, and DMN, involving the right frontal operculum and posterior part of the opercular cortex (areas OP9 and OP4), left superior and inferior parietal lobule (areas 5L; PFt [83, 84]), postcentral gyrus (left area 1, bilateral areas 2 [85] and 3b), right prosubiculum [73], and right lateral occipital cortex (area hOc5 [86]); (ii) IV_SC_, the overlapping regions primarily belonged to the visual network, SalVentAttn, and DMN, including bilateral inferior frontal gyrus (area 44), left orbitofrontal cortex (area Fo2 [87]), right mesial superior frontal gyrus (area 6ma [88]), left posterior part of the opercular cortex (area OP1 [89]), right superior parietal lobule (areas 5Ci [81, 82] and 5M), left inferior parietal lobule (area PFop [83, 84]), right precentral gyrus (areas 4a and 6d2 [69]), right ACC (area 33), and bilateral visual cortex (hOc2).

### PLSC analysis for age subgroups

#### Network-wise analysis for each age subgroup

**Figure 4.**
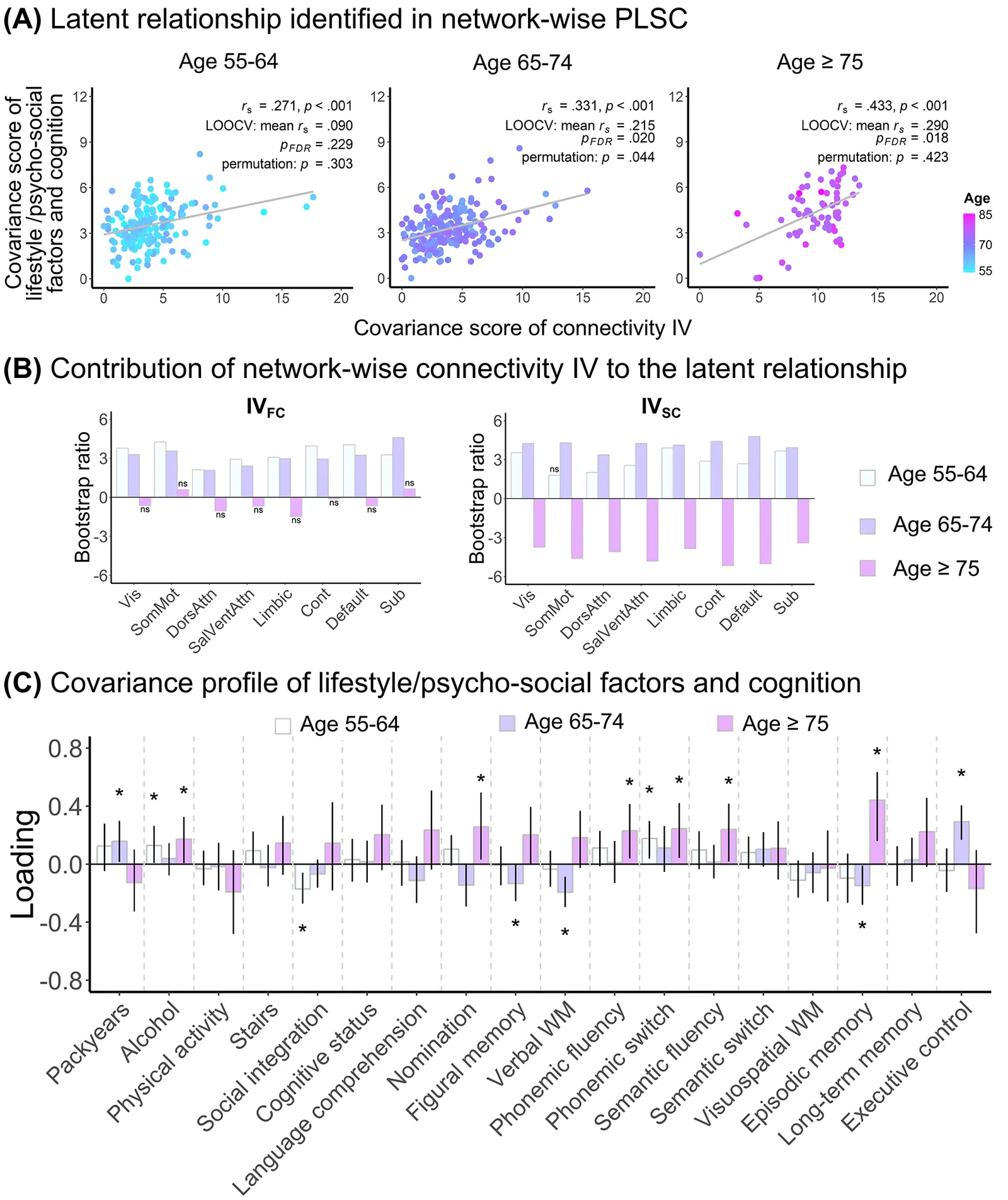
**Network-wise partial least squares correlation (PLSC) analysis (Model 1) across three age subgroups**. (**A**) Covariance scatterplots of connectivity IV (*X*-axis) vs. lifestyle/psycho-social and cognitive scores (*Y*-axis) for ages 55-64 (light blue), 65-74 (purple), and ≥75 (pink). Darker shades indicate older individuals within each group. (**B**) Bootstrap ratio bar plots for functional (IV_FC_, left) and structural connectivity (IV_SC_, right); non-significant contributions (-1.96 < ratio < 1.96) are labeled 'ns'. (**C**) Loadings of lifestyle/psycho-social and cognitive variables with 95% bootstrap confidence intervals; significant variables are marked with *. Colors match (A); faded bars indicate results not confirmed in validation analysis (e.g., age 55-64). Age, sex, and education were regressed out before analysis. Abbreviations: IV, individual variability; *r_s_*, Spearman correlation coefficient; Packyears, packyears of smoking; Alcohol, alcohol consumption; Physical activity, measured by metabolic equivalent of the task; Stairs, flights of stairs climbed; WM, working memory; Vis, visual network; SomMot, somatomotor network; DorsAttn, dorsal attention network; SalVentAttn, salience/ventral attention network; Limbic, limbic network; Cont, frontoparietal control network; Default, default mode network; Sub, subcortical network.

Network-wise Model 1 in age subgroups: combined model. In Model 1, we observed age-specific associations between lifestyle/psycho-social factors, cognitive performance, and network-wise connectivity IV (see details in Fig. 4). Supplementary Table 5 presents the loadings of lifestyle/psycho-social and cognitive variables across Models 1-3, while Supplementary Table 6 shows the bootstrap ratio scores of brain networks for all three models.

**Table 4:** Loading of the lifestyle/psycho-social factors and/or cognition covariance pattern of the region-wise partial least squares correlation (PLSC) analysis in the whole group.

	Model 1		Model 2		Model 3	
	Loading	CI (lower, upper)	Loading	CI (lower, upper)	Loading	CI (lower, upper)
Packyears	**0.11**	**0.01, 0.21**	**0.15**	**0.03, 0.25**	/	/
Alcohol	0.06	-0.02, 0.13	**0.10**	**0.02, 0.18**	/	/
MET	-0.03	-0.11, 0.07	-0.04	-0.13, 0.05	/	/
Stairs	0.01	-0.07, 0.09	0.01	-0.07, 0.10	/	/
Social integration	**-0.13**	**-0.20, -0.06**	**-0.15**	**-0.22, -0.08**	/	/
Cognitive status	-0.02	-0.12, 0.08	/	/	-0.03	-0.12,0.07
Language comprehension	-0.10	-0.21, 0.01	/	/	-0.11	-0.21, 6.80×10^-4^
Nomination	**-0.14**	**-0.23, -0.05**	/	/	**-0.14**	**-0.24, -0.06**
Figural memory	-0.08	-0.16, 4.90×10^-3^	/	/	**-0.09**	**-0.17, -3.99×10^-3^**
Verbal WM	**-0.10**	**-0.18, -0.02**	/	/	**-0.11**	**-0.19, -0.03**
Phonemic fluency	-0.02	-0.11, 0.07	/	/	-0.02	-0.11, 0.07
Phonemic switch	0.05	-0.05, 0.15	/	/	0.05	-0.06, 0.15
Semantic fluency	-0.04	-0.13, 0.04	/	/	-0.05	-0.13, 0.04
Semantic switch	0.01	-0.07, 0.10	/	/	0.01	-0.07, 0.09
Visuospatial WM	-0.03	-0.12, 0.06	/	/	-0.03	-0.12, 0.05
Episodic memory	**-0.18**	**-0.28, -0.08**	/	/	**-0.19**	**-0.29, -0.09**
Long-term memory	-0.07	-0.18, 0.02	/	/	-0.08	-0.17, 0.02
Executive control	**0.18**	**0.09, 0.28**	/	/	**0.19**	**0.10,0.29**

*Note*: A significant loading is marked as bold. Abbreviations: CI, 95% confidence intervals; Packyears, packyears of smoking; Alcohol, alcohol consumption; MET, metabolic equivalent of the task; Stairs, flights of stairs climbed; WM, working memory

For the 55-64 group, higher connectivity IV was associated with higher alcohol consumption, lower social integration, and higher performance in the phonemic switching task (*r_s_* = 0.271, *p* < 0.001). However, this relationship was not confirmed in the validation analysis (LOOCV: mean *r_s_* = 0.090, *p_FDR_* = .229; permutation: *p* = 0.303; Fig. 4A).

In the age 65-74 group, the first LV explained 73.89% of the shared variance (*p* < 0.001; Supplementary Table 4) showing a positive association between the covariance scores of brain connectivity IV and those of lifestyle/psycho-social factors and cognitive variables (*r_s_* = .331, *p* < 0.001, LOOCV: mean *r_s_* = 0.215, *p_FDR_* = 0.020; permutation: *p* = 0.044; Fig. 4A). It suggested that higher packyears of smoking and lower cognitive performance in figural memory, verbal WM, episodic memory, and executive control (see Fig. 4C and Supplementary Table 5 for variable loadings) were associated with higher connectivity IV of eight networks, particularly the IV_SC_ of the DMN (bootstrap ratio = 4.786; see Fig. 4B and Supplementary Table 6 for network contributions).

In terms of the oldest group (age ≥ 75 years), a different pattern emerged. The first LV (explained 74.10%, *p* < 0.001; see Supplementary Table 4) showed a significant correlation between covariance scores of brain connectivity IV and those of lifestyle/psycho-social and cognitive variables (*r_s_* = 0.433, *p* < 0.001; LOOCV: mean *r_s_* = 0.290, *p_FDR_* = 0.018; permutation: *p* = 0.423; Fig. 4A). Specifically, both higher alcohol consumption and higher performance in five cognitive tests were linked to lower IV_SC_ of eight networks, particularly of the frontoparietal control network (bootstrap ratio = -5.159; see Fig. 4B and Supplementary Tables 5-6). Notably, IV_FC_ showed no significant contribution.

Network-wise Model 2 in age subgroups: lifestyle/psycho-social factors only. For Model 2, however, the correlations which we observed between network-wise connectivity IV and lifestyle/psycho-social factors in the first LV was not confirmed in either of the three age subgroups (*r_s_* = .181-.448, all *p* < 0.05; LOOCV: mean *r_s_* = 0.072-.190, all *p_FDR_* > 0.05; all permutation: *p* > 0.05; see details in Supplementary Fig. 4 and Supplementary Tables 5-6).

Network-wise Model 3 in age subgroups: cognition only. In Model 3, the relationship between network-wise connectivity IV and cognition was generally consistent across the whole group and age subgroups, except for the age 55-64 group, where the association was again not confirmed (*r_s_* = 0.147, *p* = 0.044; LOOCV: mean *r_s_* = 0.089, *p_FDR_* = 0.229; permutation: *p* = 0.895; see details in Supplementary Fig. 5 and Supplementary Tables 5-6).

For the age 65-74 group (*r_s_* = 0.324, *p* < 0.001; LOOCV: mean *r_s_* = 0.230, *p_FDR_* = 0.001; permutation: *p* = 0.014), both higher IV_FC_ and IV_SC_ across the eight networks contributed, mainly linked to lower performance in figural memory, verbal WM, episodic memory, and executive control (Supplementary Fig. 5, Supplementary Tables 5-6).

In the age ≥ 75 group (*r_s_* = 0.363, *p* = 0.003; LOOCV: mean *r_s_* = 0.252, *p_FDR_* = 0.041; permutation: *p* = 0.452), only the higher IV_SC_ of the eight networks showed a significant contribution, while IV_FC_ did not. Regarding cognition, language-related variables made significant contributions (Supplementary Fig. 5, Supplementary Tables 5-6).

#### Region-wise analysis for each age subgroup

**Figure 5.**
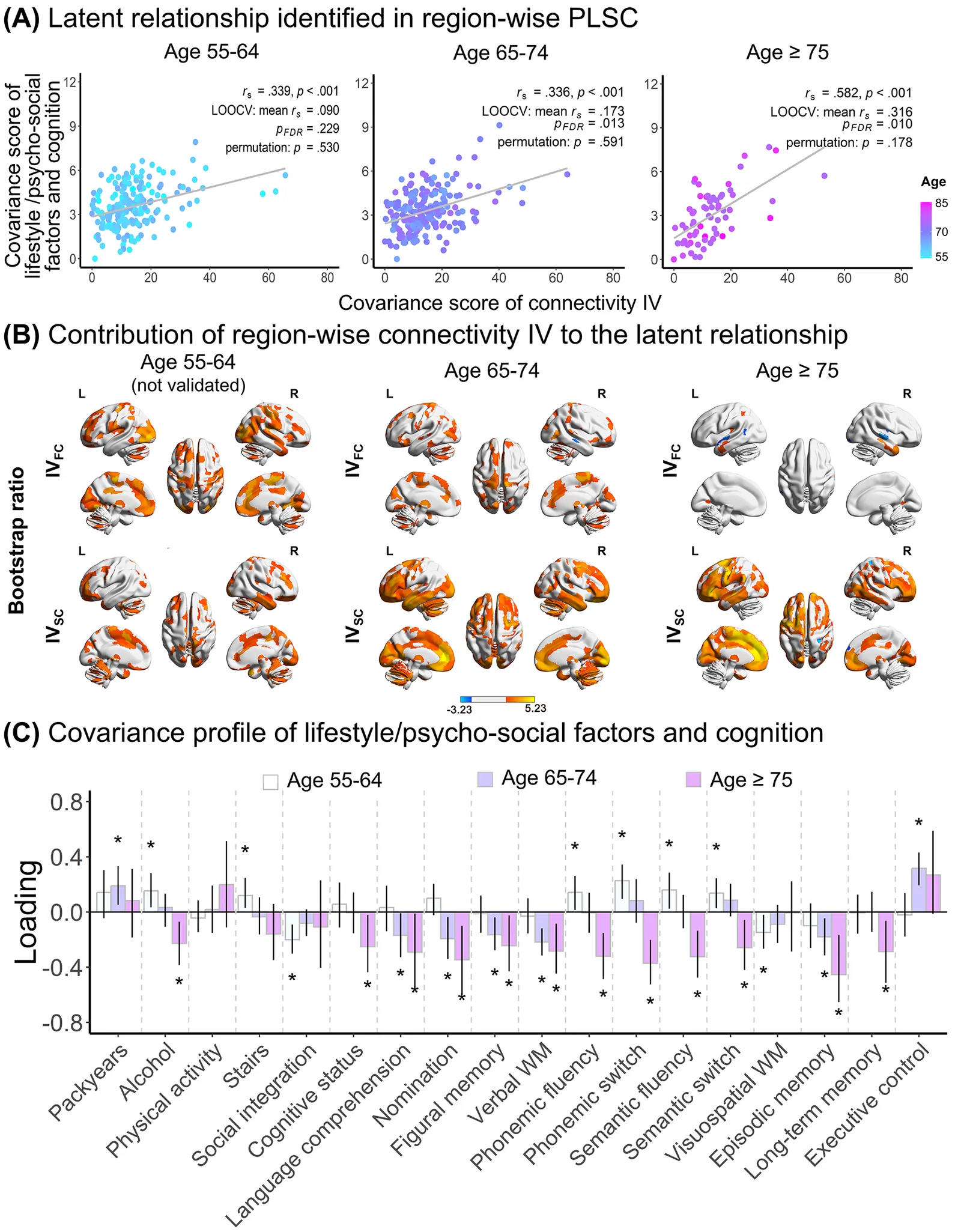
**Region-wise partial least squares correlation (PLSC) analysis (Model 1) across three age subgroups**. (**A**) Scatterplots of covariance scores: individual variability (IV) of connectivity (*X*-axis) vs. lifestyle/psycho-social and cognitive scores (*Y*-axis) for age groups 55-64 (light blue), 65-74 (purple), and ≥75 (pink). Darker shades indicate older individuals within each group. (**B**) Bootstrap ratio brain maps for region-wise IV of functional (IV_FC_, upper) and structural connectivity (IV_SC_, lower). Warm (red-yellow) and cool (blue) colors reflect positive and negative contributions, respectively. Only significant regions (|ratio| > 1.96) are shown. (**C**) Loadings of lifestyle/psycho-social and cognitive variables with 95% bootstrap confidence intervals; significant variables are marked with *. Colors match (A); faded bars indicate results not confirmed in validation analysis (e.g., age 55-64). Confounders (age, sex, education) were regressed out before PLSC. Abbreviations: IV, individual variability; *r_s_*, Spearman correlation coefficient; Packyears, packyears of smoking; Alcohol, alcohol consumption; Physical activity, measured by metabolic equivalent of the task; Stairs, flights of stairs climbed; WM, working memory.

Region-wise Model 1 in age subgroups: combined model. Region-wise analyses in Model 1 revealed age-specific patterns (see details in Fig. 5). The loadings of lifestyle/ psycho-social and/or cognitive variables (*Y* matrix) for each age group are detailed in Supplementary Table 8.

For the age 55-64 group, the correlation between region-wise connectivity IV, lifestyle/psycho-social factors, and cognition of the first LV was not confirmed (*r_s_* = 0.339, *p* < 0.001; LOOCV: mean *r_s_* = 0.090, *p_FDR_* = 0.229; permutation: *p* = 0.530; Fig. 5A).

In the age 65-74 group, a significant LV (explaining 35.93%, *p* < 0.001; see Supplementary Table 7 for model details) showed that higher connectivity IV_SC_ across widespread regions and higher IV_FC_ of several regions were associated with higher packyears of smoking and lower performance of six cognitive tests (*r_s_* = 0.336, *p* < 0.001; LOOCV: mean *r_s_* = 0.173, *p_FDR_* = 0.013; permutation *p* = 0.591; Fig. 5A, Supplementary Table 8).

For the age ≥ 75 group, the first LV (explaining 41.99%, *p* = 0.001; Supplementary Table 7) revealed that covariance scores of brain connectivity IV was significantly correlated with those of lifestyle/psycho-social and cognitive variables (*r_s_* = 0.582, *p* < 0.001; LOOCV: mean *r_s_* = 0.316, *p_FDR_* = 0.010; permutation *p* = .178; Fig. 5A). Specifically, higher region-wise connectivity IV was linked to lower alcohol consumption and lower performance in eleven cognitive tests (Fig. 5C and Supplementary Table 8). Key contributors included the IV_FC_ of a small regional cluster, i.e. the right superior temporal sulcus (area STS2), while the IV_SC_ of a large regional cluster, including the left mesial superior frontal gyrus (area 6mp [88]), frontal operculum (area OP9), precentral gyrus (area 6d2 [69]), and ACC (area p32 [90]), also made significant contributions (Fig. 5B).

Region-wise Model 2 in age subgroups: lifestyle/psycho-social factors only. For Model 2, region-wise connectivity IV correlated with lifestyle/psycho-social factors, but the associations were not confirmed for age 55-64 or 65-74 group (*r_s_* = 0.296-.330, all *p* < 0.001; LOOCV: mean *r_s_* = 0.081-.140, all *p_FDR_* > 0.05; all permutation: *p* > 0.05; see details in Figure S6). However, in the age ≥ 75 group, lower alcohol consumption and higher physical activity (i.e., metabolic expenditure) were associated with higher IV_FC_ of small regional clusters, such as the right superior parietal lobule (area 7A [81, 82]), and higher IV_SC_ across multiple regions, particularly in bilateral inferior frontal gyrus (areas 44 and 45) (*r_s_* = .628, *p* < 0.001; LOOCV: mean *r_s_* = 0.156, *p_FDR_* = 0.213; permutation *p* = 0.030; Supplementary Fig. 6, Supplementary Table 8).

Region-wise Model 3 in age subgroups: cognition only. In Model 3, the specific distribution of significantly contributing brain regions varied among age groups. No validated effect was found in the age 55-64 group (*r_s_* = 0.257, *p* < 0.001; LOOCV: mean *r_s_* = -0.089, *p_FDR_* = .229; permutation: *p* = 0.951; see details in Supplementary Fig. 7 and Supplementary Table 8). In the age ≥ 75 group (*r_s_* = 0.564, *p* < 0.001; LOOCV: mean *r_s_* = 0.295, *p_FDR_* = 0.016; permutation: *p* = 0.330; Supplementary Fig. 7, Supplementary Table 8), only small regional clusters of IV_FC_, such as the right superior temporal sulcus (area STS2), contributed significantly, compared to the age 65-74 group (*r_s_* = 0.334, *p* < 0.001; LOOCV: mean *r_s_* = 0.182, *p_FDR_* = 0.009; permutation: *p* = 0.629; Supplementary Fig. 7, Supplementary Table 8). For IV_SC_, both groups contributed significantly to a wide range of regions, especially in the left orbitofrontal cortex (area Fo6 [91]), and ACC (left area p24ab [74], bilateral area p32).

### Sensitivity analysis

After excluding outliers, we repeated both region-wise and network-wise PLSC analyses in the whole group, yielding partially consistent results with the main analysis. Specifically, in Model 1, lower social integration, along with lower performance in language comprehension, nomination, verbal WM, episodic memory, and executive control, were associated with higher region-wise connectivity IV (see Supplementary Fig. 9). Details of each LV of each model are presented in Supplementary Table 9, and the loadings of the lifestyle/psycho-social and/or cognitive variables (*Y* matrix) are provided in Supplementary Table 10. The distribution of brain regions that contributed significantly to the IV_SC_ was replicated, while only the strongest IV_FC_ contributors in the main analysis were reproduced.

In Model 2, higher packyears of smoking, more flights of stairs climbed, and lower social integration were significantly correlated with higher connectivity IV. However, this association was not confirmed (Supplementary Fig. 9, Supplementary Tables 9-10). In Model 3, lower cognitive performance in language comprehension, nomination, verbal WM, episodic memory, and executive control was linked to higher connectivity IV. The distribution of IV_SC_ of significantly contributing brain regions was almost the same as in the main analysis. However, only the IV_FC_ of brain regions with the strongest contribution in the main analysis were reproduced (Supplementary Fig. 9, Supplementary Tables 9-10).

In terms of the network-wise PLSC, the results were consistent with our main findings only for the network-wise connectivity-cognition relationship (Model 3) (see details in Supplementary Fig. 8 and Supplementary Tables 9-10; also see Supplementary Table 11 for the bootstrap ratio scores of brain networks).

## DISCUSSION

In this study, we adopted an integrative approach to investigate the intricate relationship between individual variability of functional and structural connectivity, lifestyle/psycho-social factors, and cognitive factors across a large cohort of older adults. By employing PLSC analysis for both network-wise and region-wise connectivity separately, our analysis revealed a latent variable that captured the relation between higher smoking, less social integration, lower cognitive performance, as well as higher network-wise or region-wise connectivity IV (i.e., both IV_FC_ and IV_SC_) in the whole group. In addition, the exploration of age subgroups further helped to understand the pattern of brain connectivity IV-lifestyle/psycho-social factors-cognition relationship at different stages of old age, revealing specific associations, such as the negative link between alcohol consumption and connectivity IV in the oldest group.

### Correlation between connectivity IV and cognition

The association between individual variability of the brain and cognitive functions is a relatively new research field and is therefore still to be explored. Studies, however, hint at a rather complex interplay that might be due, e.g., to the context in which it is studied. This also seems to be true across the life span; for instance, in adolescents aged 13-15, greater IV_FC_ is associated with better complex cognitive function (e.g., language reasoning) [92]. A study [17] based on data from young adults in the Human Connectome Project (HCP) (age range: 22-37 years), in contrast, reported no association between both IV_FC_ and IV_SC_ and cognition. However, in later life, individual variability of brain connectivity tends to be associated with lower cognitive performance. Specifically, Ma et al. (2021) reported a significant negative correlation between fluid intelligence, a measure of advanced cognitive ability, and connectivity IV_FC_ [15]. Although fluid intelligence is beyond the scope of this discussion, our current network-wise and region-wise analyses pointed in the same direction, i.e., that lower cognitive performance was negatively correlated with higher connectivity IV.

Critically, the interpretation of connectivity IV depends on age and its association with an outcome. Evidence indicates that all of the older subgroups (age range: 58-88 years) show higher IV_FC_ than all of the younger subgroups (age range: 18-58 years) [15], and higher IV_SC_ is also positively correlated with older age (age range: 22-68 years) [19]. Our data further indicates that there are significant differences in IV_FC_ and IV_SC_ among different age groups of older adults, with the oldest group exhibiting the highest connectivity IV (see group-level connectivity IV in Supplementary Table 3). This age-related higher connectivity IV may reflect the decreased efficiency and modularity of the brain networks [15, 93-96], as well as a decrease in the stability of inter-regional connections [97, 98], which might be one key aspect to understand its association with lower cognitive performance. Based on these observations, we assume that in adolescents, higher connectivity IV may signify adaptive plasticity, whereas in older adults, it likely reflects systemic inefficiency or network destabilization, which calls for more evidence.

In addition, according to the Posterior-to-Anterior Shift in Aging (PASA) model, as individuals age, there is a compensatory shift of activity from posterior brain regions, which are primarily involved in sensory processing, to anterior regions, particularly the prefrontal cortex [99-101]. In our results, we observed particular contributions of the connectivity IV of widespread regions in the frontal and occipital lobes to the connectivity IV-lifestyle/psycho-social factors-cognition relationship (Figure 2). This might be pointed toward older adults having less modular and less stable internal connections within the frontal and occipital lobes. If this is in accordance with PASA or might hint at an opposite mechanism of activity shift from anterior to posterior and potentially also subcortical brain regions, as assumed lately as a step-wise, experience-based process within the aging brain, counteracting the age-related PASA effects (Bilingual Anterior to Posterior and Subcortical Shift, BAPSS [102]) needs to be further elucidated.

Connectivity IV could also reflect “noise” in systems where deviations from group patterns are unrelated to functional outcomes; however, our findings that higher IV correlates with lower cognition, especially episodic memory and executive control, suggest it can be more likely attributed to inefficiency than noise. Researchers have suggested that the inter-individual variability of the brain related to specific cognitive tasks is particularly pronounced, especially in episodic memory [103, 104]. Episodic memory, which involves recalling specific details about the time and place of past events, relies on multiple regions of the brain, especially the hippocampus and the DMN [105]. Our findings align with this, as the negative correlation between episodic memory and connectivity IV was stronger than in other cognitive domains, except for executive control (Table 4). Furthermore, region-wise analysis revealed significant contributions from the IV_FC_ of the left subiculum of the hippocampal complex and both the IV_FC_ and IV_SC_ of the middle temporal gyrus. This further emphasizes that connectivity IV should be interpreted in relation to a functional outcome and that more studies are needed.

However, rather than interpreting high variability as an indication of inefficiency in brain systems and cognitive function, some studies have found that higher variability of blood-oxygen-level-dependent (BOLD) signal of brain function in older adults is associated with better performance of cognitive flexibility and processing speed in older adults [106, 107]. This contrary conclusion stems from differences in methodological definitions of variability. These studies focused on momentary intra-individual fluctuations in brain function, i.e., dynamics of functional connectivity, and this temporal variability has been interpreted as an adaptive change during the compensatory process of aging, in which more brain regions need to be recruited to counteract localized systemic degeneration [108]. Our study, however, focused on the individual variability of connectivity between regions across individuals, complementing previous research on brain heterogeneity in older adults from a different perspective.

In summary, clinical or biological explanations for individual variability of brain connectivity are complex and need to be interpreted in the context of a cognitive outcome. Future work could integrate these perspectives and compare and evaluate the results of different definitions of individual variability of brain connectivity to better understand individual heterogeneity during aging. Furthermore, future studies could also examine the variability of connectivity in clinical populations, e.g., patients suffering from Alzheimer′s Disease or other neurological diseases, to further examine the exact context in which higher or lower variability is associated with a better or worse outcome.

### How do lifestyle and psycho-social factors introduce heterogeneity of brain connectivity in older adults?

As discussed in our introduction, lifestyle and psycho-social factors may be a relevant source of higher heterogeneity in the brain in older adults. In our study, lower social integration was associated with lower cognitive performance and higher network-wise and region-wise connectivity IV. This finding is consistent with the revised scaffolding theory of aging and cognition (STAC-r) theory, which emphasizes that a rich environment, including social interaction, may strengthen the neural scaffold, while a lack of social interaction, on the other hand, might weaken it. Engaging in social activities has been shown to play a protective role in maintaining brain health and cognition during aging. For instance, James et al. (2012) found that older adults who were more socially engaged exhibited larger gray matter volumes, specifically of the temporal and occipital regions [109]. Mortimer et al. (2012) observed an increase in brain volume following a social activity intervention for older adults compared to a non-intervention group [110]. Felix et al. (2021) found that higher social engagement was significantly associated with greater gray matter microstructural integrity [111]. This was evidenced by lower mean diffusivity (MD) in regions like the bilateral lateral frontal middle gyrus, left caudate nucleus, left temporal pole, and middle temporal gyrus. A recent study by Pruitt et al. (2024) also indicated that more social engagement was associated with higher FC of the salience network in non-demented older adults, and the FC of the salience network partly mediated the association between social engagement and executive functioning and global cognition [112]. However, only a few studies have explored the relationship between social integration and brain connectivity. For example, Bittner et al.'s study (2019) of the relationship between a combined lifestyle risk score and brain structure and functional connectivity showed that social integration, smoking, and alcohol consumption drove higher functional connectivity of sensorimotor and prefrontal cortex [43].

A lifestyle factor of concern in our findings is packyears of smoking, which was associated with higher region-wise brain connectivity IV and lower cognitive performance. On one hand, smoking in older adults may contribute to individual variability of brain connectivity through various pathways. For instance, it may be associated with lower gray matter volume [113, 114] and white matter integrity [115], exacerbate accelerated brain aging [29, 116], and may link to brain connectivity in older smokers. Evidence in a study based on a large sample of 19,415 participants aged 45-78 from the UK Biobank showed that smoking was associated with lower FC in especially frontal regions [117]. Moreover, a longitudinal study found an association between frontal-insula circuits and smoking [118]. Specifically, the FC between the anterior insula and ventromedial prefrontal cortex (vmPFC) predicted long-term smoking reduction in older adults with a risk of Alzheimer’s disease.

In contrast to the brain reserve theory, which highlights factors that may counteract or decelerate age-related decline, STAC-r also takes into account the potential effects of adverse life events and factors, such as vascular disease or exposure to toxins. Drawing on this framework, smoking might be related to impaired cognitive performance, which may be indicative of fewer neural resources and less effective scaffolding compensation during aging. However, it is worth noting that we observed the effect of packyears of smoking only in the region-wise PLSC analysis, but not in the network-wise PLSC analysis. This effect of packyears of smoking in the region-wise PLSC analysis was not corroborated by the sensitivity analysis excluding outliers, though. This suggests that the observed contribution of packyears of smoking may be influenced by extreme values, indicating a subtle association rather than a robust effect. Bittner et al. (2021) also showed in their study that the association between higher packyears and higher brain age was primarily observed in moderate to severe smokers [29]. Here, it is important to consider that the packyears of smoking is a lifetime measurement, i.e., even though some of our participants may present some packyears of smoking, they were no longer smokers, which might mitigate the detrimental effects. Thus, it might be suggested that the association between smoking, cognition, and connectivity IV may vary depending on current smoking status.

According to STAC-r, a complex combination of factors associated with neural resource enrichment, as well as factors linked to less neural resource, may contribute to individual differences in the effectiveness of compensatory scaffolding. In this context, individuals with both higher packyears of smoking and lower social integration may exhibit additive or synergistic associations with fewer neural resources in the brain, potentially stronger than the associations observed for either factor alone. For example, a large-scale study [119] of adults aged over 50 years (*N* = 7,899) demonstrated a correlation between cardiovascular health scores (including diet and physical activity, blood pressure, and cholesterol) and a reduced risk of dementia. Higher cardiovascular health scores were associated with better brain health, including less hippocampal atrophy and greater brain volume. Another study (UK Biobank, *N* = 21,407, age range: 45-80 years) identified 62 brain aging components via principal component analysis with one mode-cluster linked to modifiable risk factors (e.g., smoking, alcohol consumption, and diabetes) and processing speed [120]. Compared with these studies, our study expands the focus towards the relationship between lifestyle/psycho-social factors and the brain heterogeneity of older adults, as measured by connectivity IV. As such, we observed a latent variable linking less social integration and higher packyears of smoking to higher connectivity IV and lower cognitive performance, suggesting that the accumulation of modifiable risk factors like smoking may contribute not only to lower brain structural measurements or functional connectivity but also to more complex patterns of brain aging variability. This finding aligns with emerging evidence suggesting that the accumulation of multiple modifiable risk factors in midlife is associated with differences in cognitive health, beyond the effect of individual risks [43, 121, 122]. However, causal conclusions cannot be drawn from the current cross-sectional analysis. The observed associations may be bidirectional; for example, individuals with higher connectivity IV may also be more likely to engage in fewer health-promoting lifestyle behaviors (e.g., lower social integration). Therefore, further evidence, especially from longitudinal studies, is needed.

Moreover, since Model 1 cannot directly tell which brain regions are related to lifestyle and not just driven by cognitive function, we overlapped the regions that exhibited significant contributions in both Model 1 (lifestyle/psycho-social factors and cognition) and Model 2 (lifestyle/psycho-social factors only) (see Section 3.2.2). One interpretation is that the functional or structural connectivity of these overlapping brain regions may be sensitive to factors that are associated with fewer neural resources (i.e., higher packyears of smoking and less social integration here). Such associations may reflect compromised compensatory scaffolding mechanisms in healthy older adults, which are associated with higher individual variability of connectivity and lower cognitive performance, as discussed in Section 4.1. However, these relationships should be interpreted cautiously given the cross-sectional nature of the study and the possibility of additional unmeasured confounding variables (e.g., socioeconomic status, vascular risk factors). Future research should adopt a longitudinal design to explore whether there is a potential causal relationship between connectivity IV-lifestyle/psycho-social factors, and cognition.

### Unique result patterns in different age subgroups

In the current study, we observed distinct patterns of contribution of connectivity IV across different age subgroups. Overall, with increasing age, the contribution of IV_FC_ to the model became less important to the connectivity IV-lifestyle/psycho-social factors-cognition relationship in the older group, involving fewer brain regions and smaller cluster sizes compared to IV_SC_ (Figure 5). The individual variability of structural connectivity may reflect differences in the rate and pattern of brain structural degeneration among older individuals. In older adults, brain structural connectivity is generally decreased compared to younger individuals [93, 123, 124], reflecting a directionally consistent change. This aligns with Hebling Vieira et al. (2022) [125] (*N* = 662, age range: 46-96 years), who demonstrated that combining baseline structural brain imaging data with non-brain data improved prediction of cognitive decline, whereas adding FC did not. The limited FC contribution may reflect its non-linear, variable, or dynamic nature in aging. Adaptive changes in functional connectivity would occur as compensation when white matter fibers are altered in older adults, which would then enable individuals to maintain their cognitive performance [126, 127]. In this process, FC may change in different directions [23, 95, 128, 129], i.e., higher or also lower FC with older age, making the relationship between individual variability of FC, behavior, and cognition more complex and difficult to explain by a linear model. For example, FC tends to be lower with age in the default mode, cingulo-opercular and fronto-parietal control networks, but not in the somatomotor network or the visual network, where it has been observed to be even higher with age [130]. In contrast, healthier lifestyle factors show a clearer association with larger brain volumes years later, reported in a recent work (*N* = 25,894, age range: 37-73 years) [131]. Similarly, the contribution of IV_SC_ as a structural metric, which showed greater importance with older age in our models, is likely easier to interpret with a linear model than that of IV_FC_. However, our results do not negate the predictive value of FC for cognitive function. On the contrary, we provide another perspective on the circumstances (e.g., compensation, variable and dynamic directionality) under which functional connectivity is likely to be a more challenging predictor of cognition.

Analyses for age subgroups revealed age-specific patterns of relationships between lifestyle/psycho-social factors and cognitive factors, and brain connectivity IV. In the younger group (age 55-64), while preliminary associations were observed, these did not hold under validation and should be considered as exploratory. This may indicate that the relationships between connectivity IV and cognition and/or lifestyle/psycho-social factors are not similarly present in this age-subgroup as compared to the older age groups, or too subtle to detect with the current methodology. Specifically, one may assume that the transitional nature of this age subgroup, from working to retirement, may contribute to higher heterogeneity in factors such as physical health, psychosocial conditions, and environmental exposures, which were not assessed in the current study and may obscure early or subtle effects. Furthermore, age-related changes in brain and behavior may follow a nonlinear trajectory, with more pronounced and measurable effects emerging after age 65, and may be more sensitive to accumulated risk factors, such as lifestyle factors. Therefore, while these results do not provide robust evidence for associations in this age subgroup, further research with larger samples and a longitudinal design is needed to clarify these patterns.

For the age 65-74 group, we found that higher packyears of smoking, combined with lower cognition (e.g., figural memory, verbal WM, episodic memory, and executive control), were associated with both higher network-wise and region-wise connectivity IV, which were consistent with that of the whole group. Inconsistently, the contribution of social integration was not significant in the age 65-74 group and the oldest age group. It cannot be excluded that this is due to the relatively small sample size reducing the statistical power, making it harder to detect significant effects compared to the analysis of the whole group. For the age ≥ 75 years group, the negative associations of alcohol consumption and cognitive performance with network-wise and region-wise IV_SC_ instead of IV_FC_ highlight the potential vulnerability of this age group to structural connectivity decline. Unlike relatively younger age groups, where functional connectivity may compensate for structural impairments, the oldest participants may exhibit reduced flexibility of FC and can no longer counteract SC decline, as suggested by previous studies [132]. Overall, these findings suggest that the brain connectivity IV-lifestyle/psycho-social factors-cognition relationship dynamically changes with age, which typically is not focused on in group studies. Age stratification should be considered in population studies to decipher the relationships between variables of different ages in more depth.

Numerous studies have shown that cognitive abilities tend to decline more rapidly in older people who consume alcohol than in those who do not [133-136]. For instance, one study highlighted that older adults with drinking habits are at a higher risk of cognitive impairment, suggesting that long-term alcohol consumption negatively affects cognitive function [133], despite hypotheses have suggested that light-to-moderate drinking [137, 138] or red wine consumption [139, 140] might benefit the brain. Our results observed that alcohol consumption and cognitive performance negatively correlated with IV_FC_ and IV_SC_ in the oldest group, i.e., higher alcohol consumption was associated with lower connectivity IV and better cognitive function. Given the reported prior literature, the direction of this result is counter-intuitive since one would expect higher alcohol consumption to be related to lower cognitive performance. One possible explanation we considered was whether this relationship is due to a generally lower drinking behavior of the oldest group compared to the other two groups, such that higher alcohol consumption would in reality reflect a slight drinking pattern, which was not the case in our sample, see Table 1. Thus, a general lower alcohol consumption in the oldest group does not explain the observed results. Another possible explanation might be that the oldest participants who were still capable of participating in the examination might be those individuals who had successfully maintained good physical and cognitive health, such that overall effects of alcohol consumption might have been compensated for, i.e. they might present other protective factors, such as cognitive reserve (e.g., high socioeconomic status or high education) [141]. In addition, older individuals who subjectively perceive cognitive decline may follow medical advice to reduce adverse behaviors, such as alcohol consumption, which could therefore lead to a reverse relationship between drinking, brain connectivity IV, and cognition. However, the observed correlation might also be overestimated due to the relatively small sample size in the oldest group (*n* = 66) [142]. Thus, this observation is likely driven by methodological limitations, such as small sample size, sample characteristics, or additional influence of covariates, hence not allowing a conclusion of the general benefit of light drinking in very old participants. Indeed, there is evidence that an increased risk of cognitive decline in older adults is associated with any level of drinking [143].

In summary, compared with previous large-scale studies that primarily focused on the relationship between unimodal [113, 131] or multimodal [115-117] brain metrics and single lifestyle factors or a composite lifestyle score [29, 43], the current study offers a different perspective. We uniquely explored the relationship between (i) multimodal connectivity IV and (ii) cognitive performance, as well as the triangular relation with (iii) multiple lifestyle and psycho-social factors in healthy older adults. Furthermore, while a few large-scale multimodal studies [120, 125] have investigated similar triangular relationships, the current study specifically advances our understanding of aging heterogeneity by focusing on connectivity IV and integrating these three dimensions. E.g., prior studies observing social integration as a factor mostly focused on one specific brain metric (e.g., either gray matter volume or white matter integrity) and seldom integrated various modalities. Here, we found a latent variable linking higher IV_FC_ and IV_SC_ to lower social integration and more packyear of smoking and lower cognitive performance, and thus, offering a more systemic insight. We also expanded evidence for connectivity IV from infants [16, 18], adolescents [92], as well as young [14, 17, 144] and middle-aged adults [9] to healthy older adults aged ≥ 55 years and further refined the patterns of results in specific age subgroups. Critically, IV_FC_ and IV_SC_ help illuminate relationships with cognition and/or lifestyle that are obscured when ignoring individual variability. However, even existing studies on IV_FC_ [2, 15] or IV_SC_ [19] in older adults examined variability metrics but overlooked systematic links to lifestyle factors. By linking multimodal connectivity IV to lifestyle/psycho-social factors and cognition, the current study adds empirical evidence supporting the STAC-r theory and offers theoretical support for developing personalized interventions targeting cognitive decline in aging populations.

### Limitations

Several limitations of this work need to be acknowledged to ensure accurate interpretation of the results.

The omission of regressing out the potential effect of intra-individual variability of connectivity (obtained by calculating the dissimilarity between multiple scans of individuals over a short period of time), from the inter-individual variability of connectivity, as seen in previous studies [2, 15, 17, 19, 144], poses a constraint. Some researchers [18, 145] have split the data from one session into two halves to calculate the intra-individual variability over time, which is removed as noise. However, we retained full scan durations and avoided splitting the data to maintain robust signal-to-noise ratio (SNR) and statistical power in functional connectivity estimates, as shorter time-series segments are known to reduce reliability [146, 147]. Therefore, we cannot directly compare the distribution pattern of brain connectivity IV with previous studies on it (see group-level connectivity IV in Supplementary Table 3). The replication of the findings from the current study in an external cohort, such as the UK Biobank, should be considered in future studies.

Furthermore, the current sample consisted of a relatively homogeneous cohort of German older adults regarding education (education level = 6.39±1.95; 50.8% held a high school degree with qualification to study at a university, “Abitur” in German). While this homogeneity may reduce variation introduced by confounding sociocultural factors and enhance statistical power for detecting effects within this specific population, it may limit the generalizability of the observed results to more diverse populations [148]. Lifestyle factors may have different relationships with cognition and the brain across populations with varying cultural, ethnic, and educational backgrounds [149]. Especially the tight link between education and cognition makes it desirable to further study the association with lifestyle and connectivity IV in educationally diverse cohorts, which should be addressed in future research.

The unequal sample sizes in each age subgroup, especially the relatively small sample of 66 participants in the age ≥ 75 years group, may impact the reliability of the results. A study [142] suggested that studies with small sample sizes tend to have larger effect sizes than those with large samples. This is not only relevant for the discussion related to alcohol consumption but may generally explain why we are more likely to observe an effect in the age ≥ 75 years group or a more significant effect compared to other age groups. Future studies should consider a larger sample size in this age group to enhance the reliability of the results. In addition, the lifestyle and psycho-social factors measured in this study are self-reported, which is a typical practice for these types of population-based studies [28, 150]. While self-reported measures may be prone to errors such as social desirability bias and recall bias, self-report measurements have nevertheless been shown to be valid and reliable [151].

The cross-sectional design of this study limits its capacity to establish causative effects. Although certain lifestyle behaviors (e.g., packyears of smoking) tend to be relatively stable over time, we cannot definitively determine whether unhealthy lifestyle/psycho-social factors contribute to a higher brain connectivity IV or lower performance of cognition, or vice versa. Future studies are encouraged to adopt longitudinal designs to better understand the interplay between lifestyle/psycho-social factors, cognition, and individual variability of brain connectivity during aging. In addition, only age, sex, and education were statistically controlled in this study. Other potentially important confounding factors, such as genetic predispositions, general physical health, socioeconomic status, vascular risk factors, psychiatric history, and medication use, were not included, but may influence the findings as well, which could be addressed in future studies. Finally, studies should also be extended to more diverse populations and explore additional lifestyle factors (e.g., sleep, diet) to develop a more comprehensive and inclusive understanding of the neural mechanisms underlying individual differences in aging.

### Conclusion

Overall, our findings linked brain heterogeneity to lifestyle/psycho-social factors and cognitive performance, suggesting that higher network-wise and region-wise individual variability of connectivity is associated with lower social integration and/or higher smoking, and lower cognitive performance, such as nomination, episodic memory, and executive control in a large sample of older adults. IV_FC_ and IV_SC_ of several regions were significantly related not only to cognition but also to lifestyle/psycho-social factors. The result patterns of this relationship varied by age group. Individual variability of connectivity was positively correlated with smoking in the age 65-74 group and negatively correlated with alcohol consumption in the oldest group. IV_SC_ tends to contribute more than IV_FC_ to this relationship across ages. These findings provide empirical evidence for the STAC-r theory, suggesting that an unhealthy lifestyle and less social integration may be associated with higher individual variability of connectivity in older adults, which may involve differences in neural resources and be linked to cognitive decline. However, future research is needed to further elucidate the causal relationships among these factors.

## Supplementary Materials

The Supplementary data can be found online at: www.aginganddisease.org/EN/10.14336/AD.2025.0428.


