# Supplementary material for: Linking Individual Variability of Multi-modal Connectivity, Lifestyle, Psycho-Social Factors and Cognition in Older Adults
Source: Aging Dis. 2026 Jul 22;17(5):2683–709. doi: 10.14336/AD.2025.0428 (PMC13437116; doi:10.14336/AD.2025.0428)
Supplement: Supplementary file 1 [file AD-17-5-2683-s.pdf]

SUPPLEMENTARY DATA

Linking Individual Variability of Multi-modal Connectivity, Lifestyle, Psycho-Social Factors and Cognition in Older Adults

Mingxian Zhang, Susanne Moebus, Nico Dragano, Nora Bittner, Svenja Caspers

© 2025. Cao R et al. Published online at http://www.aginganddisease.org/EN/10.14336/AD.2025.0428

SUPPLEMENTARY DATA

eMETHODS

1. eMethods 1: Neuropsychological tests description

Details about thirteen Neuropsychological tests are listed in Supplementary Table1.

2. eMethods 2: Data preprocessing

Preprocessed data were derived from the previous study [1]. Details of the visual image quality checks as well as the exclusion criteria for MRI images can be seen in [1]. Specifically, for diffusion-weighted imaging (DWI) data, visual quality control was performed after eddy current and motion artifacts correction. Sub-optimal volumes or datasets were removed from further analyses due to ghosting, remaining signal dropouts, or very noisy data (n = 69).

For functional MRI data, ICA-based Automatic Removal Of Motion Artifacts (ICA-AROMA) [2] was applied to identify and remove motion-related independent components from functional MRI data. Then, AROMA was combined with global signal regression in the current study. Finally, all resting-state fMRI images were band-pass filtered (0.01–0.1 Hz) and registered to the standard space template (MNI152) using the unified segmentation approach [3]. With AROMA, particularly focusing on the correction of intensity artifacts induced by head motion, we further took advantage of an established algorithm by Afyouni and Nichols (2018) [4] to check for each participant's volume-wise severe intensity dropouts by generating p values for spikes (DVARS) on preprocessed functional data. If participants with more than 10% of the 300 volumes [1, 5] were detected to be missing, they were excluded (n = 8) from further analysis. Further, based on the preprocessed mean AROMA functional data, we checked for potential misalignments by performing the "check sample homogeneity using standard deviation across sample" function provided by the CAT12 toolbox [6] and excluded participants for which the individual image did not align to the MNI152 template (>2 SD, n = 8).

3. eMethods 3: Measuring the connectivity IV

Group-level connectivity IV

Group-level individual variability of connectivity (connectivity IV) was computed according to definitions and formulas from previous studies [7, 8]. For each ROI, region i, the individual similarity (IS) was assessed by calculating the average correlation between all pairs of FC or SC vectors, excluding self-correlations. Taking the FC as an example, the IS_{FCi} is calculated as follows:

$IS_{FCi} = E[corr(FC_{i}\_m, FC_{i}\_n]$

where $m, n = 1, 2, \dots 461$, and $m \neq n$.

FC_{i}_m/n represents the FC of region i in the brain for a particular participant m or n. It is denoted as a 1×461 correlation coefficient vector, in which each element corresponds to the correlation between the FC of region i and the FC of all other regions for the participant m or n.

And then individual variability of FC (IV_{FC}) at region i was calculated by its inverted similarity (1-IS_{FCi}) [7]:

$IV_{FCi} = 1- IS_{FCi}$ ($i = 1, 2, \dots 421$)

This calculation was repeated for all regions to derive the spatial distribution of the individual variability (a 421 × 1 vector) of the functional connectivity across the entire brain. The individual variability of SC (IV_{SCi}) was calculated in a similar way. To perform a network-wise analysis, we

© 2025. Cao R et al. Published online at http://www.aginganddisease.org/EN/10.14336/AD.2025.0428

averaged the IV_{FC} and IV_{SC} values of 421 brain regions across eight networks separately (see results in eResults 2).

4. eMethods 4: Partial least squares correlation

The specific process of PLSC analysis is as follows:

1) *X*-*Y* correlation calculation

The behavioral-brain correlation matrix was calculated. The smaller dimension of the correlation matrix equals the latent variable (LV) (*j*) count. Take Model 1 of region-wise analysis as an example. X matrix includes 842 features of region-wise brain connectivity IV while the *Y* matrix consists of 18 features of lifestyle/psycho-social factors and cognition variables. In this case, this was the number of lifestyle/psycho-social factors and cognition variables ($j = 18$).

2) Singular value decomposition (SVD)

Singular value decomposition was performed on the *X*-*Y* correlation matrix which resulted in a set of mutually orthogonal LVs. Singular value decomposition results in a left singular vector matrix (U: 842×18), right singular vector matrix (V: 18×18), and a diagonal matrix of singular values (18×18). Together, these represent a set of latent variables with a LV being composed of a left and right singular vector and a corresponding singular value.

3) Extraction of latent variables

Each LV represents a specific covariance pattern within the input data. A singular vector weight the corresponding original variables to maximize their covariance, i.e., the weighted regional values of a singular vector (U, LV j) can be interpreted as a maximally covarying brain individual variability pattern and its corresponding lifestyle/psycho-social factors and cognition pattern (V, LV j).

4) Explained Variance Calculation

The explained variance of a LV was calculated as the ratio of its corresponding squared singular value to the sum of the remaining squared singular values. The significance of a LV was assessed by comparing the observed explained variance to a non-parametric distribution of permuted values acquired by randomly permuting the subject order in ($N = 5{,}000$).

5) Subject-Specific Covariance Scores

Subject-specific scores measure to which extent an individual expresses a covariance pattern represented by a LV. Thus, scores can be thought of as factor weightings in factor analysis. A high score describes the high agreement of a participant with the identified pattern. They were calculated by projecting U on *X* for covariance scores of brain connectivity IV (UX), and V on Y for a covariance score of lifestyle/psycho-social factors and cognition (VY).

6) Bootstrap resampling

Bootstrap resampling was performed to identify brain connectivity IV and lifestyle/psycho-social factors and cognition variables with a high and robust contribution to the *X*-*Y* association. Individuals were randomly sampled from and with replacement ($N = 5{,}000$) which resulted in a set of resampled correlation matrices propagated to singular value decomposition resulting in a sampling distribution of singular vector weights for each input variable. This enabled the computation of 95% confidence intervals (CI) for the lifestyle/psycho-social factors and cognition variables and a bootstrap ratio for the brain regions. The bootstrap ratio measures a brain region's contribution to the observed covariance pattern of a respective LV, as a relevant region shows a high singular vector weight alongside a small standard error implying stability across bootstraps [9, 10].

© 2025. Cao R et al. Published online at http://www.aginganddisease.org/EN/10.14336/AD.2025.0428

SUPPLEMENTARY DATA

Bootstrap ratio = Singular vector weight *U* (regions *i*, LV *j*)/ Standard error estimated from bootstrapping

eRESULTS

1. eResults 1: Neuropsychological measurements

Supplementary Table 2 lists the characteristics of neuropsychological measurements in the whole group as well as three age subgroups. Except for phonemic fluency, 12 cognitive domains showed significant differences among three age subgroups.

2. eResults 2: Characteristics of group-level inter-individual variability of connectivity

Supplementary Table 3 lists the characteristics of group-level individual variability of connectivity in the whole group as well as three age subgroups. Except for the IV_{SC} of the dorsal attention network (DorsAttn), salience/Ventral attention network (SalVentAttn), and Subcortical network (Sub), connectivity IV was significantly different among the three age subgroups.

3. eResults 3: Partial least squares correlation (PLSC)
4. Network-wise PLSC analysis

Whole group
Supplementary Table 4 lists the LV of each model in network-wise analysis for the whole group and three age subgroups.
Supplementary Figure 2 shows the results of network-wise Model 2 and Model 3 in the whole group.
Age subgroups
Supplementary Table 5 lists the loading of the lifestyle/psycho-social factors and cognition covariance pattern of the network-wise PLSC analysis in three age subgroups.
Supplementary Table 6 lists the bootstrap ratio score in network-wise analysis for three age subgroups.
For Model 2, results indicated that higher alcohol consumption and lower social integration were associated with higher IV_{FC} of 6 networks (insignificant in the DorsAttn, and limbic network) and higher IV_{SC} of 7 networks (insignificant in the limbic network). For Model 3, consistent with the results of Model 1, lower performance in four cognitive domains was associated with higher IV_{FC} of 6 networks and IV_{SC} of 8 networks.
Supplementary Figure 4-5 show the results of network-wise Model 2 and Model 3 among the three age subgroups.
For Model 2, overall, the correlations between lifestyle/psycho-social factors and connectivity IV were not confirmed across the three age subgroups. For Model 3, the correlation between network-wise connectivity IV and cognition was not confirmed in the age 55-64 group. In the other two groups, we observed differences in the specific contribution of the network to the covariance score of the connectivity IV. Specifically, in the age 65-74 group, lower performance in four cognitive domains was associated with higher IV_{FC} and IV_{SC} of 8 networks. In the age ≥75 group, IV_{SC} of 8 networks - but not IV_{FC} - was significantly associated with lower performance in 4 language-related domains as well as episodic memory.
© 2025. Cao R et al. Published online at http://www.aginganddisease.org/EN/10.14336/AD.2025.0428

5. Region-wise PLSC analysis

Whole group

Supplementary Table 7 lists the LV of each model in the region-wise PLSC analysis for the whole group and three age subgroups.

Supplementary Figure 3 shows the results of region-wise Model 2 and Model 3 in the whole group.

Age subgroups

Supplementary Table 8 lists the loading of lifestyle/psycho-social factors and/or cognition variables in the region-wise PLSC analysis for three age subgroups.

Supplementary Figure 6-7 show the results of region-wise Model 2 and Model 3 among the three age subgroups.

For Model 2, the correlation between region-wise connectivity IV and lifestyle/psycho-social factors of the 1st LV was only validated in the age ≥75 group, showing that lower alcohol consumption, higher physical activity (measured by metabolic equivalent of the task [MET]) were associated with higher IVFC of the small region of occipital lobe and left posterior cingulate gyrus. For the Model 3, relations between cognition and connectivity IV were validated in the age 65-74 group and the age ≥75 group.

6. eResults 4: Sensitivity analysis

Supplementary Table 9 lists the results of network-wise PLSC for Models 1-3 in 403 participants.

Supplementary Figure 8 shows the results of network-wise Models 1-3 in 403 participants.

The correlations between the *X* matrix and *Y* matrix were validated in Model 1(combined) and Model 3 (cognition only), but not in Model 2 (lifestyle/psycho-social factors only). For Model 1, the results showed that lower performance in nomination, episodic memory, and executive control were associated with higher connectivity IV of 8 networks. For Model 3, lower performance in the same three cognitive domains was associated with higher IV_{FC} of six networks and IV_{SC} of eight networks.

Supplementary Table 10-11 list the results of region-wise PLSC for Models 1-3 in 403 participants.

Supplementary Figure 9 shows the results of region-wise Model 1-3 in 403 participants.

Again, the correlations between the X matrix and Y matrix were validated in Model 1(combined) and Model 3 (cognition only), but not in Model 2 (lifestyle/psycho-social factors only). For Model 1, the results showed that lower social integration and lower performance in 5 cognitive domains were associated with higher IV_{FC} of regions such as the thalamus, anterior cingulate gyrus, and higher IV_{SC} of widespread brain regions. For Model 3, the results pattern seems similar to Model 1.

© 2025. Cao R et al. Published online at http://www.aginganddisease.org/EN/10.14336/AD.2025.0428

Supplementary Table 1. Description of neuropsychological measurements.

Variables	Test (References)	Cognitive domain	Description
Cognitive status	DemTect [11]	Cognitive functioning	The sum of the scores from a digit span memory test, a number conversion task, and a verbal fluency task (sum score).
Language comprehension	Wortschatztest (Word-hoard-test) [12]	General language comprehension, Vocabulary	Total number of target words correctly identified among interfering words (sum score).
Nomination	Boston Naming Test (from CERAD; short form) [13]	Nomination	The number of correct answers on an object-naming task (using frequent, medium-frequent, and rare items) (sum score).
Figural memory	Benton-Test [14]	Visual memory, figural memory	Total number of correctly free recalled figures from 20 previously presented figures (sum score).
Verbal WM	Zahlennachsprechen (from Nürnberger Alters-Inventar) [15]	Verbal working memory	Total number of correctly recalled digits given in a backward sequence (sum score).
Phonemic fluency	Regensburger Wortflüssigkeitstest [16]	Semantic verbal fluency	Total number of produced words beginning with a given letter B in 2 minutes (sum score).
Phonemic switch	Regensburger Wortflüssigkeitstest [16]	Semantic verbal switch	Total number of produced words beginning with a given letter G-R in 2 mins (sum score).
Semantic fluency	Regensburger Wortflüssigkeitstest [16]	Semantic verbal fluency	Total number of produced words in the category “Berufe (job)” in 2 mins (sum score).
Semantic switch	Regensburger Wortflüssigkeitstest [16]	Semantic switch	Total number of produced words in the category “sports-fruits” in 2 mins (sum score).
Visuospatial WM	Corsi block tapping task (CBT) [17]	Visual- spatial working memory	The number of correct answers on a backward block sequence recall task (with gradually increasing sequence length) (sum score).
Episodic memory	Bielefelder Kategorielle Wortlisten (BKW) [18]	Verbal learning and memory capabilities	The average of the relative proportions of reproduced free recalls across five runs.
Long-term memory	Bielefelder Kategorielle Wortlisten (BKW) [18]	Verbal long-term memory	Total number of correctly free recalled words from a list of 15 words in delayed run across 5 trials (sum score).
Executive control	Trail making test (TMT) A and B (taken from CERAD-Plus) [13]	Cognitive flexibility, concept shifting	Time difference between completion time (sec.) of TMT Part B and completion time of TMT Part A (TMTB-A).

Note: Further descriptions are also found in Caspers et al., 2014 [19].

© 2025. Cao R et al. Published online at http://www.aginganddisease.org/EN/10.14336/AD.2025.0428

Supplementary Table 2. Neuropsychological measurements of the participants.

	Mean (*SD*)				Statistic	
	Whole group (*N* = 461)	Age 55-64 (*n* = 189)	Age 65-74 (*n* = 206)	Age 75- 85 (*n* = 66)	χ^2/F-value	*p*-value
Cognitive status [a]	14.86 (2.34)	15.24 (2.36)	14.88 (2.26)	13.73 (2.24)	17.411	**.001**
Language comprehension [a]	30.95 (4.79)	31.47 (4.22)	31.13 (4.86)	28.92 (5.58)	13.133	**.001**
Nomination [a]	14.53 (0.76)	14.69 (0.61)	14.53 (0.78)	14.09 (0.91)	30.336	**<.001**
Figural memory [a]	8.70 (4.06)	10.36 (3.67)	8.00 (3.98)	6.09 (3.40)	66.762	**<.001**
Verbal WM [a]	4.7 (1.07)	4.87 (1.16)	4.61 (1.00)	4.52 (0.93)	9.604	**.008**
Phonemic fluency [a]	18.97 (6.20)	19.36 (6.13)	18.80 (5.86)	18.39 (7.39)	1.627	.443
Phonemic switch [b]	18.63 (5.96)	19.29 (5.46)	18.50 (5.94)	17.15 (7.08)	3.259	**.039**
Semantic fluency [a]	23.21 (6.90)	23.99 (6.73)	23.25 (6.94)	20.83 (6.77)	9.254	**.010**
Semantic switch [a]	19.66 (4.67)	20.29 (4.34)	19.66 (4.62)	17.88 (5.33)	15.112	**<.001**
Visuospatial WM [a]	4.70 (1.08)	4.92 (1.05)	4.52 (1.09)	4.61 (1.05)	13.173	**.001**
Episodic memory [b]	60.11 (11.69)	64.11 (10.52)	58.36 (11.31)	54.08 (12.30)	24.39	**<.001**
Long-term memory [a]	10.74 (2.68)	11.4 (2.51)	10.60 (2.55)	9.30 (2.93)	32.678	**<.001**
Executive control [a]	54.31 (38.59)	43.39 (25.18)	55.57 (36.03)	81.67 (59.16)	50.619	**<.001**

Note: The mean value and standard deviation are resented as *mean* (*SD*). Statistical analysis performed among three age subgroups.

[a] Independent-Samples Kruskal-Wallis Test

[b] One way ANOVA

Abbreviations: WM, working memory; TMT, trail making test

Supplementary Table 3. Characteristics of group-level connectivity IV.

	Mean (SD)				Statistic	
Group-level IV	Whole group (*N* = 461)	Age 55-64 (*n* = 189)	Age 65-74 (*n* = 206)	Age ≥ 75 (*n* = 66)	*F*-value	*p*-value
**IV_{FC}**						
whole brain	0.71 (.08)	0.69 (0.09)	0.70 (0.09)	0.77 (0.07)	115.103	**<.001**
Vis	0.70 (0.07)	0.67 (0.07)	0.70 (0.07)	0.75 (0.05)	26.083	**<.001**
SomMot	0.75 (0.09)	0.65 (0.05)	0.66 (0.06)	0.75 (0.04)	98.363	**<.001**
DorsAttn	0.68 (0.06)	0.71 (0.07)	0.72 (0.07)	0.78 (0.06)	17.245	**<.001**
SalVentAttn	0.71 (0.06)	0.68 (0.08)	0.68 (0.09)	0.75 (0.06)	13.157	**<.001**
Limbic	0.68 (0.09)	0.83 (0.05)	0.83 (0.05)	0.86 (0.03)	4.688	**.012**
Cont	0.69 (0.08)	0.71 (0.06)	0.71 (0.06)	0.77 (0.06)	17.530	**<.001**
Default	0.70 (0.08)	0.66 (0.09)	0.66 (0.10)	0.74 (0.08)	25.035	**<.001**
Sub	0.72 (0.14)	0.80 (0.05)	0.81 (0.05)	0.85 (0.06)	4.346	**.020**
**IV_{SC}**						
whole brain	0.37 (0.09)	0.35 (0.09)	0.36 (0.09)	0.40 (0.10)	29.509	**<.001**
Vis	0.36 (0.10)	0.31 (0.07)	0.32 (0.07)	0.36 (0.09)	6.627	**.002**
SomMot	0.34 (0.08)	0.38 (0.06)	0.39 (0.06)	0.42 (0.08)	4.795	**.009**
DorsAttn	0.36 (0.06)	0.35 (0.06)	0.37 (0.07)	0.39 (0.08)	2.343	.100
SalVentAttn	0.38 (0.08)	0.39 (0.11)	0.41 (0.11)	0.45 (0.13)	2.950	.056
Limbic	0.36 (0.05)	0.29 (0.06)	0.31 (0.06)	0.35 (0.06)	7.981	**<.001**
Cont	0.36 (0.08)	0.33 (0.07)	0.35 (0.07)	0.38 (0.09)	6.718	**.002**
Default	0.38 (0.11)	0.34 (0.10)	0.36 (0.10)	0.39 (0.10)	7.330	**<.001**
Sub	0.46 (0.10)	0.44 (0.12)	0.47 (0.13)	0.51 (0.14)	1.176	.319

Note: The mean value and standard deviation are resented as mean (*SD*). The between-group differences among age subgroups were examined by one way ANOVA. A significant *p*-value is marked as bold. Abbreviations: FC/IV_{SC}, individual variability of functional/ structural connectivity; Vis, visual network; SomMot, somatomotor network; DorsAttn, dorsal attention network; SalVentAttn, salience/ventral attention network; Limbic, limbic network; Cont, frontoparietal control network; Default, default mode network; Sub, subcortical network

© 2025. Cao R et al. Published online at http://www.aginganddisease.org/EN/10.14336/AD.2025.0428

Supplementary Table 4. Network-wise PLSC analysis of three models: explained variance and *p*-values of latent variables in the whole group and three age subgroups.

Latent variable	Whole group		Age 55-64		Age 65-74		Age ≥75	
	Explained variance (%)	*p*-value	Explained variance (%)	*p*-value	Explained variance (%)	*p*-value	Explained variance (%)	*p*-value
**Model 1**								
1	73.89	**<.001**	50.42	**.001**	71.17	**<.001**	74.10	**<.001**
2	11.41	.176	31.17	**.009**	14.16	**.041**	15.36	**.010**
3	5.93	.260	6.43	.237	4.51	.321	3.96	.590
4	2.27	.625	3.20	.569	3.21	.737	1.94	.730
5	1.65	.803	2.29	.672	1.63	.820	1.22	.940
6	1.55	.834	1.88	.827	1.46	.811	1.03	.960
7	0.91	.984	1.48	.911	0.99	.985	0.77	.970
8	0.57	.996	1.08	.975	0.89	.971	0.46	1
9	0.54	.991	0.75	.984	0.56	.995	0.39	.990
10	0.44	.994	0.45	1	0.45	1	0.29	1
11	0.40	1	0.34	1	0.44	.997	0.16	1
12	0.17	1	0.24	1	0.27	1	0.12	1
13	0.11	1	0.12	1	0.14	1	0.07	1
14	0.09	1	0.11	1	0.08	1	0.06	1
15	0.04	1	0.02	1	0.04	1	0.03	1
16	0.01	1	0.01	1	0.01	1	0.01	1
**Model 2**								
1	76.46	**.002**	67.36	**.004**	73.44	**.013**	68.79	**.023**
2	14.77	.360	22.95	.093	10.22	.675	21.72	.323
3	4.39	.890	5.77	.720	7.71	.827	4.31	.954
4	3.31	.976	2.53	.958	5.27	.969	3.25	.981
5	1.06	1	1.40	.998	3.36	.987	1.92	.999
**Model 3**								
1	74.35	**<.001**	46.98	**.018**	76.93	**<.001**	77.71	**.001**
2	13.76	.243	33.35	**.027**	10.97	.215	14.25	**.016**
3	4.50	.469	8.04	.196	4.59	.456	2.52	.754
4	2.59	.637	4.11	.462	2.78	.823	2.22	.757
5	1.65	.821	2.29	.873	1.77	.867	0.98	.976
6	1.11	.962	1.95	.932	1.21	.833	0.69	.985
7	0.70	.988	1.65	.934	0.70	.984	0.61	.981
8	0.47	.999	0.66	.990	0.43	.999	0.41	.998
9	0.34	1	0.40	.999	0.36	.997	0.26	1
10	0.23	1	0.25	1	0.18	1	0.18	1
11	0.15	1	0.19	1	0.08	1	0.11	1
12	0.12	1	0.12	1	0.01	1	0.05	1
13	0.04	1	0.01	1	0.00	1	0.00	1

Note: A significant *p*-value is marked as bold.

© 2025. Cao R et al. Published online at http://www.aginganddisease.org/EN/10.14336/AD.2025.0428

Supplementary Table 5. Network-wise PLSC analysis of three models: loading of the lifestyle/psycho-social and cognitive variables in three age subgroups.

Variables	Age 55-64 Loading	Age 55-64 CI (lower,upper)	Age 65-74 Loading	Age 65-74 CI (lower,upper)	Age ≥75 Loading	Age ≥75 CI (lower,upper)
**Model 1**						
Packyears	0.13	-0.05,0.28	**0.16**	**0.02,0.30**	-0.13	-0.33,0.10
Alcohol	**0.13**	**0.01,0.26**	0.04	-0.08,0.14	**0.17**	**0.01,0.33**
MET	-0.03	-0.15,0.09	-0.02	-0.18,0.15	-0.19	-0.48,0.10
Stairs	0.09	-1.15×10^{-3},0.23	-0.02	-0.15,0.13	0.15	-0.07,0.33
Social integration	**-0.17**	**-0.27,-0.06**	-0.07	-0.16,0.03	0.14	-0.18,0.43
Cognitive status	0.03	-0.12,0.17	0.02	-0.13,0.16	0.20	-0.04,0.41
Language comprehension	0.02	-0.15,0.17	-0.11	-0.27,0.05	0.24	-0.04,0.51
Nomination	0.10	-0.01,0.20	-0.14	-0.29,0.01	**0.26**	**0.03,0.49**
Figural memory	-0.01	-0.15,0.12	**-0.13**	**-0.25,-3.99×10^{-3}**	0.20	-2.65×10^{-3},0.39
Verbal WM	-0.03	-0.16,0.09	**-0.19**	**-0.30,-0.09**	0.18	-0.03,0.37
Phonemic fluency	0.11	-0.01,0.23	0.01	-0.13,0.16	**0.23**	**0.04,0.41**
Phonemic switch	**0.18**	**0.04,0.30**	0.11	-0.05,0.26	**0.24**	**0.04,0.42**
Semantic fluency	0.10	-0.03,0.23	0.01	-0.10,0.13	**0.24**	**0.02,0.42**
Semantic switch	0.08	-0.03,0.19	0.10	-0.02,0.22	0.11	-0.11,0.29
Visuospatial WM	-0.11	-0.23,0.02	-0.06	-0.20,0.08	-0.03	-0.26,0.23
Episodic memory	-0.10	-0.27,0.07	**-0.15**	**-0.28,-0.01**	**0.44**	**0.16,0.63**
Long-term memory	-1.45×10^{-3}	-0.15,0.12	0.03	-0.12,0.18	0.22	-0.02,0.46
Executive control	-0.04	-0.19,0.11	**0.29**	**0.17,0.40**	-0.17	-0.48,0.10
**Model 2**						
Packyears	0.17	-2.88×10^{-3},0.32	**0.19**	**0.06,0.32**	0.14	-0.06,0.34
Alcohol	**0.13**	**0.01,0.25**	0.04	-0.10,0.14	**-0.19**	**-0.35,-0.04**
MET	-0.03	-0.15,0.11	-0.01	-0.19,0.17	0.21	-0.08,0.50
Stairs	0.09	-1.23×10^{-3},0.22	-0.05	-0.16,0.08	-0.15	-0.34,0.07
Social integration	**-0.17**	**-0.28,-0.05**	**-0.10**	**-0.19,-4.40×10^{-3}**	-0.19	-0.47,0.14
**Model 3**						
Cognitive status	0.08	-0.06,0.22	0.02	-0.12,0.17	-0.20	-0.40,0.04
Language comprehension	0.04	-0.10,0.17	-0.12	-0.28,0.05	-0.24	-0.51,0.04
Nomination	**0.11**	**0.01,0.19**	-0.14	-0.28,0.01	**-0.26**	**-0.50,-0.02**
Figural memory	0.02	-0.12,0.14	**-0.14**	**-0.25,-0.01**	-0.20	-0.39,0.01
Verbal WM	0.01	-0.12,0.14	**-0.20**	**-0.31,-0.09**	-0.18	-0.36,0.03
Phonemic fluency	0.12	-0.01,0.24	0.02	-0.12,0.17	**-0.23**	**-0.43,-0.04**
Phonemic switch	**0.21**	**0.07,0.32**	0.12	-0.04,0.27	**-0.25**	**-0.43,-0.04**
Semantic fluency	0.10	-0.05,0.24	0.02	-0.10,0.14	**-0.24**	**-0.42,-0.02**
Semantic switch	0.08	-0.04,0.19	0.11	-0.01,0.22	-0.11	-0.30,0.11
Visuospatial WM	-0.06	-0.18,0.08	-0.05	-0.19,0.08	0.03	-0.22,0.25
Episodic memory	-0.05	-0.22,0.12	**-0.16**	**-0.28,-0.02**	**-0.44**	**-0.63,-0.17**
Long-term memory	0.05	-0.08,0.16	0.03	-0.11,0.18	-0.22	-0.45,0.01
Executive control	-0.04	-0.19,0.11	**0.29**	**0.17,0.40**	0.17	-0.10,0.47

Note: A significant loading is marked as bold. Abbreviations: CI, 95% confidence intervals; Packyears, packyears of smoking; Alcohol, alcohol consumption; MET, metabolic equivalent of the task; Stairs, flights of stairs climbed; WM, working memory

© 2025. Cao R et al. Published online at http://www.aginganddisease.org/EN/10.14336/AD.2025.0428

Supplementary Table 6. Network-wise PLSC analysis of three models: bootstrap ratio score of brain networks in three age subgroups.

Networks	**IV_{FC}** Age 55-64	Age 65-74	Age ≥75	**IV_{SC}** Age 55-64	Age 65-74	Age ≥75
**Model 1**						
Vis	**3.781**	**3.291**	-0.635	**3.537**	**4.244**	**-3.755**
SomMot	**4.241**	**3.553**	0.587	1.800	**4.307**	**-4.600**
DorsAttn	**2.117**	**2.076**	-1.053	**2.011**	**3.374**	**-4.099**
SalVentAttn	**2.916**	**2.407**	-0.680	**2.557**	**4.253**	**-4.82**
Limbic	**3.069**	**2.963**	-1.477	**3.905**	**4.128**	**-3.845**
Cont	**3.954**	**2.940**	-0.128	**2.875**	**4.409**	**-5.159**
Default	**4.036**	**3.238**	-0.659	**2.683**	**4.786**	**-5.012**
Sub	**3.26**	**4.589**	0.641	**3.660**	**3.923**	**-3.412**
**Model 2**						
Vis	**2.760**	-0.481	1.863	**2.261**	**2.906**	1.831
SomMot	**3.734**	-0.030	-0.860	0.665	**3.035**	**3.582**
DorsAttn	**2.852**	0.451	0.096	0.538	**2.065**	**4.882**
SalVentAttn	**3.080**	0.199	-0.817	**2.045**	**3.085**	**4.19**
Limbic	**3.494**	-0.874	1.051	**3.179**	**2.684**	1.641
Cont	**3.849**	0.461	0.238	1.706	**3.103**	**4.073**
Default	**3.436**	0.613	-1.001	**2.385**	**3.376**	**4.129**
Sub	1.880	0.019	0.716	**2.374**	**2.882**	**1.973**
**Model 3**						
Vis	**2.395**	**3.669**	0.057	**3.256**	**3.888**	**3.532**
SomMot	1.708	**3.788**	-0.445	1.901	**3.758**	**3.810**
DorsAttn	0.156	**2.141**	1.133	**2.204**	**3.009**	**3.369**
SalVentAttn	0.799	**2.637**	0.902	**2.043**	**3.866**	**3.915**
Limbic	1.114	**3.470**	1.362	**3.711**	**3.918**	**3.866**
Cont	1.679	**3.079**	0.095	**3.072**	**4.083**	**4.480**
Default	1.818	**3.399**	0.969	**2.574**	**4.452**	**4.237**
Sub	**2.688**	**4.889**	-0.906	**3.375**	**3.420**	**3.180**

Note: A significant bootstrap ratio is marked as bold. Abbreviations: $IV_{FC/SC}$, individual variability of functional/ structural connectivity; Vis, visual network; SomMot, somatomotor network; DorsAttn, dorsal attention network; SalVentAttn, salience/ventral attention network; Limbic, limbic network; Cont, frontoparietal control network; Default, default mode network; Sub, subcortical network

© 2025. Cao R et al. Published online at http://www.aginganddisease.org/EN/10.14336/AD.2025.0428

Supplementary Table 7. Region-wise PLSC analysis of three models of three models: explained variance and *p*-values of latent variables in the whole group and three age subgroups.

Latent variable	**Whole group** Explained variance (%)	*p*-value	**Age 55-64** Explained variance (%)	*p*-value	**Age 65-74** Explained variance (%)	*p*-value	**Age ≥75** Explained variance (%)	*p*-value
**Model 1**								
1	40.06	**<.001**	28.50	**.001**	35.93	**<.001**	41.99	**.001**
2	13.75	.179	22.49	**.004**	11.89	.392	12.40	**.016**
3	6.73	.324	8.56	.142	8.55	.314	7.94	.399
4	5.83	.275	6.55	.329	6.59	.106	6.23	.632
5	4.71	.511	5.00	.400	5.80	.463	4.80	.444
6	3.97	.683	4.00	.962	3.99	.940	4.38	.724
7	3.31	.875	3.27	.929	3.70	.732	3.70	.693
8	3.00	.988	3.07	.975	3.50	.867	3.48	.944
9	2.79	.989	2.80	.915	3.31	.605	2.61	.982
10	2.51	.979	2.64	.988	2.86	.950	2.35	.982
11	2.40	.984	2.31	.940	2.73	.997	2.22	.994
12	2.18	.999	2.15	.997	2.35	.985	1.91	.989
13	2.07	.998	2.08	.953	2.07	.904	1.41	.982
14	1.86	.971	1.80	1	1.80	.999	1.37	.992
15	1.59	.997	1.62	1	1.63	1	1.09	.992
16	1.52	1	1.51	1	1.51	.999	1.00	.984
**Model 2**								
1	47.41	**.001**	44.71	**.004**	42.28	**.011**	37.88	**.021**
2	18.03	.466	24.71	.051	18.36	.643	21.45	.273
3	14.57	.798	12.52	.520	15.27	.916	15.94	.747
4	1.74	.990	1.54	.993	13.51	.731	14.65	.711
5	9.25	.995	7.51	.990	1.58	.999	1.08	.926
**Model 3**								
1	43.57	**<.001**	29.56	.051	41.77	**<.001**	5.57	**.002**
2	17.60	.202	26.51	**.008**	14.66	.646	12.56	.055
3	7.62	.492	9.08	.091	1.15	.172	7.46	.348
4	6.16	.530	6.55	.288	6.90	.389	6.00	.862
5	4.58	.889	5.98	.842	5.82	.633	5.31	.448
6	4.08	.629	4.37	.923	4.39	.931	3.75	.944
7	3.43	.985	3.92	.794	3.40	.778	3.62	.888
8	2.94	.941	3.32	.897	3.00	.984	2.92	.986
9	2.88	.940	3.02	.987	2.87	.840	2.12	.985
10	2.46	.954	2.82	.998	2.47	.929	1.86	.998
11	2.27	.999	2.25	1	1.99	.999	1.64	.993
12	1.22	.997	1.36	1	1.35	.998	1.44	.891
13	1.19	1	1.26	1	1.25	.993	0.75	.997

Note: A significant *p*-value is marked as bold.

© 2025. Cao R et al. Published online at http://www.aginganddisease.org/EN/10.14336/AD.2025.0428

Supplementary Table 8. Region-wise PLSC analysis of three models: loading of the lifestyle/psycho-social and/or cognitive variables in three age subgroups.

	**Age 55-64**		**Age 65-74**		**Age ≥75**	
	Loading	CI (lower,upper)	Loading	CI (lower,upper)	Loading	CI (lower,upper)
**Model 1**						
Packyears	0.14	-0.04,0.30	**0.19**	**0.05,0.33**	0.08	-0.18,0.31
Alcohol	**0.15**	**0.04,0.28**	0.03	-0.11,0.13	**-0.23**	**-0.38,-0.07**
MET	-0.04	-0.15,0.08	0.02	-0.15,0.19	0.20	-0.11,0.51
Stairs	**0.12**	**0.03,0.25**	-0.04	-0.16,0.11	-0.16	-0.35,0.06
Social integration	**-0.20**	**-0.30,-0.09**	-0.08	-0.17,0.02	-0.11	-0.40,0.23
Cognitive status	0.06	-0.11,0.21	-0.01	-0.15,0.14	**-0.25**	**-0.44,-0.02**
Language comprehension	0.03	-0.14,0.19	**-0.17**	**-0.33,-0.01**	**-0.29**	**-0.55,-0.01**
Nomination	0.10	-0.02,0.20	**-0.19**	**-0.34,-0.04**	**-0.35**	**-0.60,-0.10**
Figural memory	-0.01	-0.15,0.12	**-0.16**	**-0.28,-0.04**	**-0.24**	**-0.43,-0.03**
Verbal WM	-0.03	-0.15,0.10	**-0.22**	**-0.31,-0.12**	**-0.28**	**-0.45,-0.08**
Phonemic fluency	**0.14**	**0.02,0.26**	-0.01	-0.15,0.14	**-0.32**	**-0.49,-0.15**
Phonemic switch	**0.23**	**0.09,0.34**	0.08	-0.08,0.24	**-0.37**	**-0.53,-0.20**
Semantic fluency	**0.16**	**0.03,0.28**	1.53×10^{-4}	-0.12,0.12	**-0.32**	**-0.48,-0.14**
Semantic switch	**0.14**	**0.03,0.24**	0.09	-0.03,0.20	**-0.26**	**-0.42,-0.06**
Visuospatial WM	**-0.15**	**-0.27,-0.02**	-0.09	-0.23,0.05	-0.01	-0.29,0.22
Episodic memory	-0.10	-0.26,0.06	**-0.18**	**-0.31,-0.05**	**-0.45**	**-0.65,-0.17**
Long-term memory	-0.01	-0.16,0.12	-1.35×10^{-3}	-0.14,0.15	**-0.29**	**-0.51,-0.06**
Executive control	-0.02	-0.18,0.14	**0.32**	**0.19,0.43**	0.27	-0.01,0.59
**Model 2**						
Packyears	**0.22**	**0.01,0.40**	**0.31**	**0.2,0.41**	0.19	-0.01,0.4
Alcohol	**0.15**	**0.04,0.27**	0.05	-0.11,0.16	**-0.36**	**-0.54,-0.17**
MET	-0.05	-0.16,0.08	-0.04	-0.20,0.13	**0.38**	**0.08,0.60**
Stairs	**0.13**	**0.03,0.24**	-0.10	-0.21,0.03	-0.14	-0.33,0.08
Social integration	**-0.23**	**-0.34,-0.12**	**-0.15**	**-0.23,-0.05**	-0.22	-0.52,0.12
**Model 3**						
Cognitive status	**0.20**	**0.06,0.33**	-4.28×10^{-3}	-0.15,0.14	**-0.25**	**-0.44,-0.01**
Language comprehension	0.11	-0.04,0.24	**-0.18**	**-0.33,-0.01**	**-0.30**	**-0.55,-0.01**
Nomination	**0.12**	**0.03,0.20**	**-0.19**	**-0.34,-0.04**	**-0.36**	**-0.62,-0.12**
Figural memory	0.09	-0.05,0.20	**-0.17**	**-0.28,-0.04**	**-0.25**	**-0.44,-0.04**
Verbal WM	0.09	-0.05,0.23	**-0.23**	**-0.33,-0.13**	**-0.27**	**-0.43,-0.07**
Phonemic fluency	**0.19**	**0.07,0.30**	1.59×10^{-3}	-0.14,0.15	**-0.34**	**-0.52,-0.17**
Phonemic switch	**0.30**	**0.17,0.41**	0.09	-0.06,0.24	**-0.39**	**-0.55,-0.22**
Semantic fluency	**0.18**	**0.02,0.32**	0.01	-0.11,0.13	**-0.34**	**-0.50,-0.16**
Semantic switch	**0.14**	**0.03,0.25**	0.09	-0.03,0.21	**-0.28**	**-0.44,-0.08**
Visuospatial WM	-0.03	-0.15,0.11	-0.09	-0.23,0.06	-6.46×10^{-3}	-0.29,0.22
Episodic memory	0.03	-0.13,0.18	**-0.19**	**-0.32,-0.06**	**-0.46**	**-0.65,-0.18**
Long-term memory	0.10	-0.02,0.21	-0.01	-0.15,0.14	**-0.30**	**-0.52,-0.08**
Executive control	-0.06	-0.21×0.10	**0.32**	**0.19,0.44**	**0.28**	**1.26×10^{-3},0.60**

Note: A significant loading is marked as bold. Abbreviations: CI, 95% confidence intervals; Packyears, packyears of smoking; Alcohol, alcohol consumption; MET, metabolic equivalent of the task; Stairs, flights of stairs climbed; WM, working memory

© 2025. Cao R et al. Published online at http://www.aginganddisease.org/EN/10.14336/AD.2025.0428

Supplementary Table 9. Sensitivity analysis: latent variables of PLSC analysis in 403 participants.

Latent variable	Model 1 Explained variance (%)	*p*-value	Model 2 Explained variance (%)	*p*-value	Model 3 Explained variance (%)	*p*-value
**Network-wise**						
1	68.30	**<.001**	70.20	**.010**	70.84	**<.001**
v	14.34	.083	21.15	.320	14.10	.235
3	7.52	.417	5.30	.966	7.31	.351
4	2.48	.759	2.39	.997	2.54	.831
5	2.29	.729	0.96	1	1.53	.843
6	1.46	.889	/	/	1.29	.953
7	1.07	.987	/	/	0.91	.992
8	0.82	.978	/	/	0.60	1
9	0.55	.999	/	/	0.39	1
10	0.44	1	/	/	0.20	1
11	0.30	1	/	/	0.14	1
12	0.20	1	/	/	0.07	1
13	0.13	1	/	/	0.06	1
14	0.07	1	/	/	/	/
15	0.03	1	/	/	/	/
16	0.01	1	/	/	/	/
**Region-wise**						
1	33.80	**<.001**	35.03	**.018**	39.94	**.001**
2	13.72	.150	21.19	.323	16.63	.283
3	7.21	.115	20.22	.479	8.64	.272
4	6.30	.369	12.63	.916	6.86	.803
5	5.54	.789	10.95	.990	5.40	.700
6	4.78	.913	/	/	4.25	.641
7	3.88	.663	/	/	3.77	.990
8	3.32	.862	/	/	3.57	.963
9	3.20	.984	/	/	3.17	.969
10	3.08	.921	/	/	2.64	.990
11	2.73	.995	/	/	2.44	.999
12	2.70	.966	/	/	1.42	.982
13	2.27	.932	/	/	1.27	1
14	2.11	.998	/	/	/	/
15	1.75	1	/	/	/	/
16	1.66	1	/	/	/	/
17	1.02	.985	/	/	/	/
18	0.91	1	/	/	/	/

Note: A significant *p*-value is marked as bold.

© 2025. Cao R et al. Published online at http://www.aginganddisease.org/EN/10.14336/AD.2025.0428

Supplementary Table 10. Sensitivity analysis: loading of the lifestyle/psycho-social factors and/or cognition covariance pattern of the PLSC analysis in 403 participants.

Variables	**Model 1**		**Model 2**		**Model 3**	
	Loading	CI (lower,upper)	Loading	CI (lower,upper)	Loading	CI (lower,upper)
**Network-wise**						
Packyears	0.07	-0.04,0.19	0.08	-0.03,0.20	/	/
Alcohol	0.02	-0.06,0.11	0.03	-0.06,0.12	/	/
MET	0.01	-0.10,0.13	3.48×10^{-4}	-0.11,0.11	/	/
Stairs	0.07	-0.02,0.16	0.08	-0.02,0.17	/	/
Social integration	-0.10	-0.18,9.21×10^{-4}	**-0.10**	**-0.18,-0.01**	/	/
Cognitive status	-0.04	-0.14,0.06	/	/	-0.04	-0.14,0.06
Language comprehension	-0.09	-0.20,0.03	/	/	-0.08	-0.19,0.02
Nomination	**-0.10**	**-0.20,-0.01**	/	/	**-0.10**	**-0.20,-0.01**
Figural memory	-0.05	-0.14,0.05	/	/	-0.05	-0.14,0.04
Verbal WM	-0.09	-0.18,0.01	/	/	-0.08	-0.17,0.01
Phonemic fluency	-0.01	-0.11,0.09	/	/	-0.01	-0.11,0.08
Phonemic switch	0.04	-0.06,0.14	/	/	0.04	-0.07,0.14
Semantic fluency	-0.02	-0.12,0.08	/	/	-0.02	-0.12,0.08
Semantic switch	0.04	-0.06,0.13	/	/	0.04	-0.05,0.13
Visuospatial WM	-0.01	-0.10,0.09	/	/	-3.31×10^{-3}	-0.10,0.09
Episodic memory	**-0.14**	**-0.25,-0.03**	/	/	**-0.14**	**-0.25,-0.03**
Long-term memory	-0.02	-0.12,0.08	/	/	-0.01	-0.12,0.09
Executive control	**0.16**	**0.06,0.27**	/	/	**0.16**	**0.06,0.27**
**Region-wise**						
Packyears	0.08	-0.03,0.20	**0.12**	**0.01,0.23**	/	/
Alcohol	1.83×10^{-3}	-0.08,0.09	0.05	-0.04,0.14	/	/
MET	0.02	-0.09,0.15	0.01	-0.09,0.11	/	/
Stairs	0.06	-0.03,0.15	**0.13**	**0.03,0.23**	/	/
Social integration	**-0.10**	**-0.19,-0.01**	-0.13	-0.21,-0.04	/	/
Cognitive status	-0.05	-0.15,0.06	/	/	-0.05	-0.15,0.05
Language comprehension	**-0.13**	**-0.24,-0.01**	/	/	**-0.14**	**-0.25,-0.03**
Nomination	**-0.15**	**-0.25,-0.05**	/	/	**-0.16**	**-0.27,-0.06**
Figural memory	-0.07	-0.16,0.02	/	/	-0.07	-0.16,0.01
Verbal WM	**-0.10**	**-0.19,-0.01**	/	/	**-0.10**	**-0.19,-0.01**
Phonemic fluency	-0.04	-0.14,0.06	/	/	-0.05	-0.14,0.05
Phonemic switch	0.01	-0.10,0.11	/	/	7.59×10^{-4}	-0.11,0.10
Semantic fluency	-0.05	-0.15,0.05	/	/	-0.05	-0.15,0.04
Semantic switch	0.01	-0.08,0.11	/	/	0.01	-0.08,0.10
Visuospatial WM	-0.02	-0.11,0.08	/	/	-0.02	-0.11,0.08
Episodic memory	**-0.17**	**-0.27,-0.06**	/	/	**-0.18**	**-0.28,-0.06**
Long-term memory	-0.05	-0.15,0.06	/	/	-0.05	-0.16,0.05
Executive control	**0.19**	**0.09,0.30**	/	/	**0.19**	**0.09,0.31**

Note: A significant loading is marked as bold. Abbreviations: CI, 95% confidence intervals; Packyears, packyears of smoking; Alcohol, alcohol consumption; MET, metabolic equivalent of the task; Stairs, flights of stairs climbed; WM, working memory

© 2025. Cao R et al. Published online at http://www.aginganddisease.org/EN/10.14336/AD.2025.0428

Supplementary Table 11. Sensitivity analysis: bootstrap ratio of network-wise PLSC analysis in 403 participants.

Networks	**IV_{FC}**			**IV_{SC}**		
	Model 1	Model 2	Model 3	Model 1	Model 2	Model 3
Vis	**3.859**	**2.333**	**3.221**	**3.987**	1.838	**3.571**
SomMot	**3.453**	**2.357**	**2.610**	**3.770**	1.142	**3.541**
DorsAttn	1.956	1.606	1.434	**3.367**	0.941	**3.202**
SalVentAttn	1.431	1.508	0.895	**4.101**	**2.155**	**3.480**
Limbic	**2.773**	1.232	**2.559**	**3.405**	1.743	**2.965**
Cont	**3.120**	**2.881**	**2.236**	**3.806**	**2.182**	**3.270**
Default	**2.672**	1.797	**2.133**	**3.683**	**2.165**	**3.051**
Sub	**3.457**	**2.590**	**2.697**	**3.904**	**2.656**	**3.359**

Note: A significant bootstrap ratio is marked as bold. Abbreviations: $IV_{FC/SC}$, individual variability of functional/ structural connectivity; Vis, visual network; SomMot, somatomotor network; DorsAttn, dorsal attention network; SalVentAttn, salience/ventral attention network; Limbic, limbic network; Cont, frontoparietal control network; Default, default mode network; Sub, subcortical network

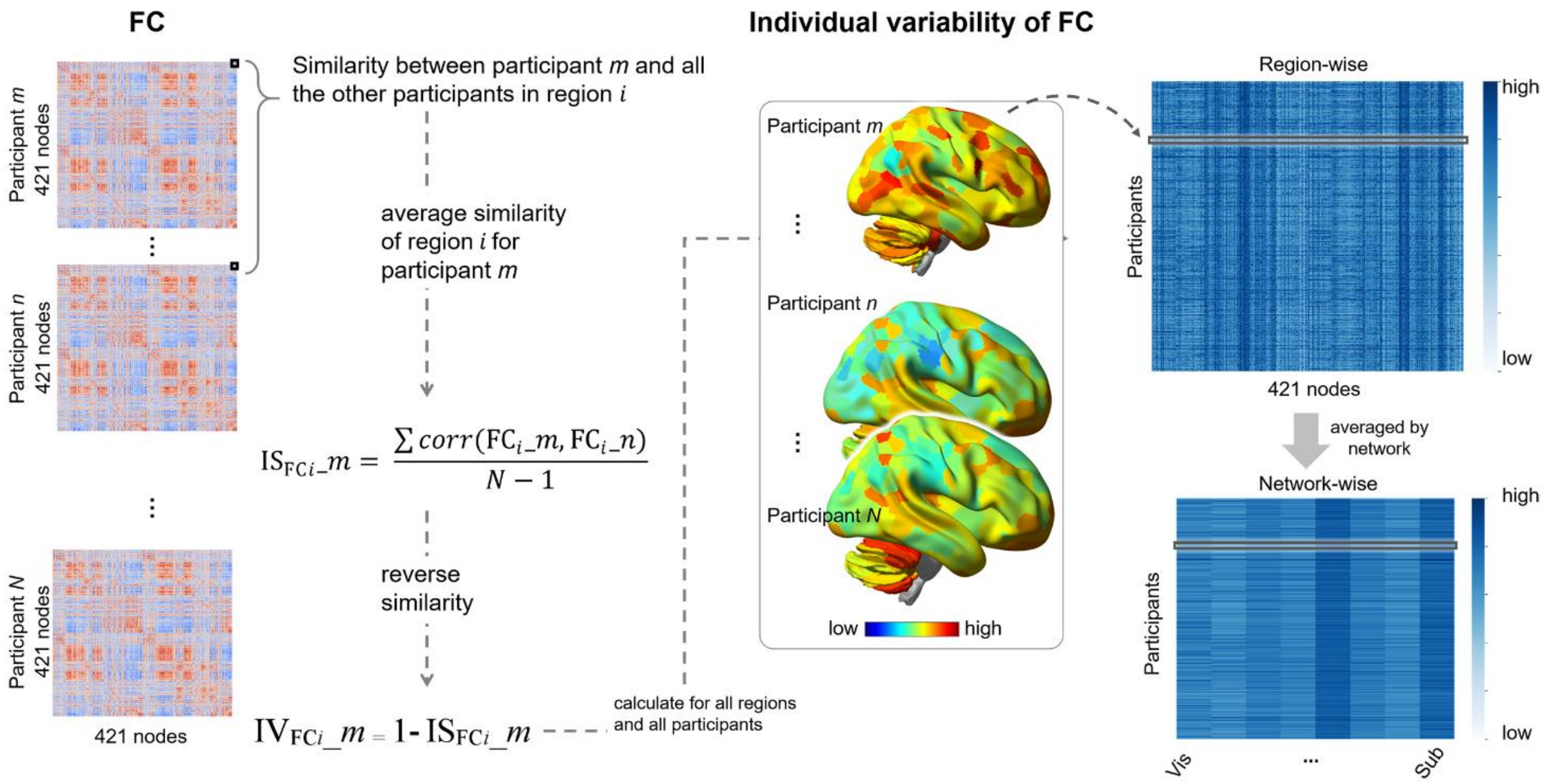

Supplementary Figure 1. Calculation of connectivity IV. Here, we use functional connectivity (FC) as an example to illustrate the calculation of individual variability (IV) in connectivity. First, individual similarity of FC (IS_{FC}) was computed for each participant *m* as the average correlation between *m*'s FC and the FCs of all other participants (*N*-1) at each brain region *i* (*i* = 1, 2, …, 421). Second, the similarity value for participant m at region *i* (i.e., IV_{SCi}_m) was transformed into individual variability (IV_{FCi}_m) by converting similarity (ranging from −1 to 1) into dissimilarity (ranging from 0 to 2). Higher IV_{FC} values indicate greater dissimilarity between an individual's FC pattern at region *i* and the group-average FC pattern for that region. Finally, IV_{FC} was computed across all regions and participants, resulting in an IV_{FC} matrix of size 461×421. Network-wise IV_{FC} was then obtained by averaging the IV_{FC} values across regions within each network (matrix of size 461×8).

© 2025. Cao R et al. Published online at http://www.aginganddisease.org/EN/10.14336/AD.2025.0428

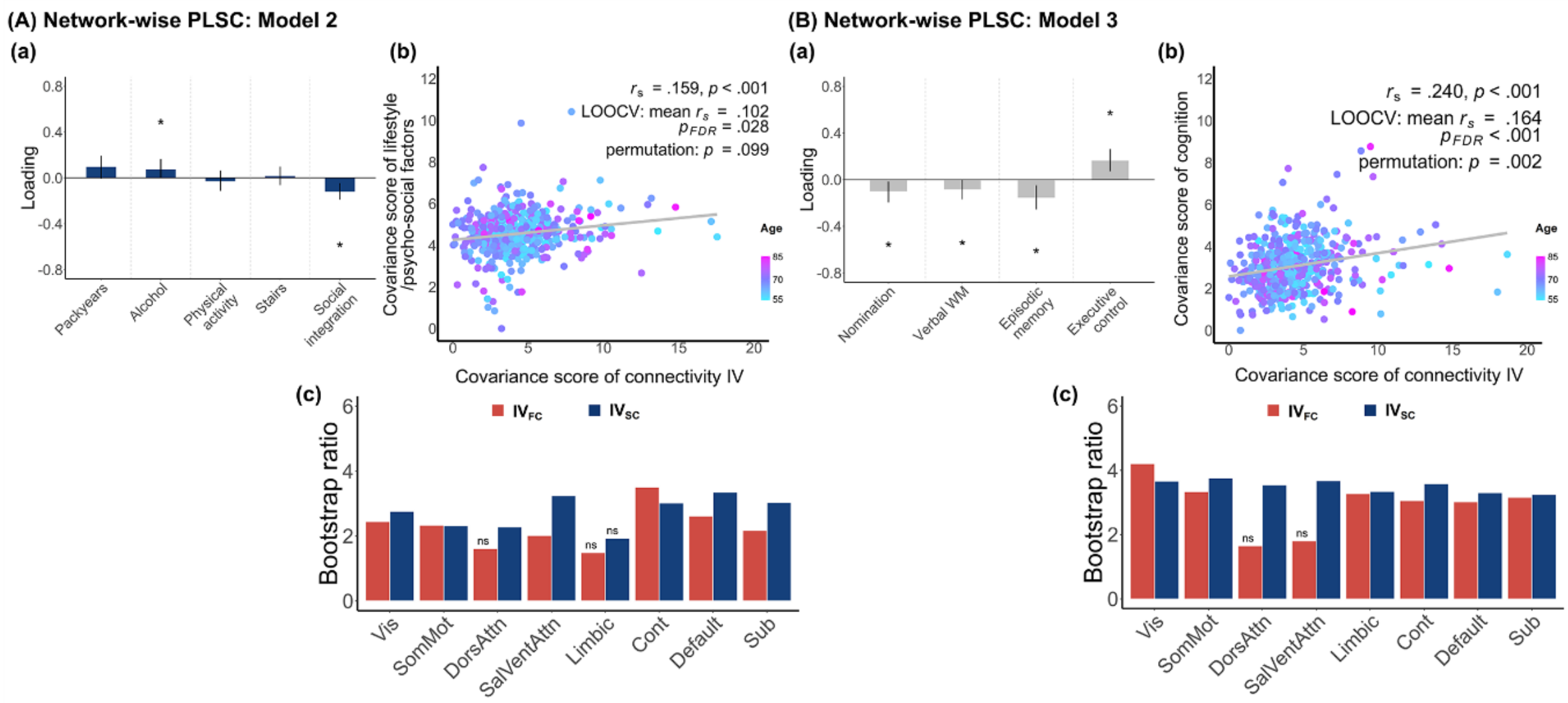

Supplementary Figure 2. Model 2 (A) and Model 3 (B) of network-wise PLSC analysis in the whole group. The bar plots show the loading of lifestyle/psycho-social factors [A (a)] or cognition [B (a)], with 95% confidence intervals calculated via bootstrap resampling. Significant contributors are marked with an asterisk (*). A scatter plot shows the correlation between covariance scores of individual variability (IV) of connectivity (*X*-axis) and covariance scores (*Y*-axis) of lifestyle/psycho-social factors [A (b)] or cognition [B (b)], with participant ages color-coded (55 to 85 years old, light blue to pink). The bootstrap ratio bar charts [A (c) and B (c)] represent the covariance patterns of network-wise IV of functional (IV_{FC}, red) and structural connectivity (IV_{SC}, blue). Positive/negative ratios indicate positive/negative contributions to the overall pattern. 'ns' denotes non-significant ratios (−1.96 < ratio < 1.96). Confounders (age, sex, education) were regressed out before PLSC. Abbreviations: r_s, Spearman correlation coefficient; Packyears, packyears of smoking; Alcohol, alcohol consumption; Physical activity, measured by metabolic equivalent of the task; Stairs, flights of stairs climbed; WM, working memory; Vis, visual network; SomMot, somatomotor network; DorsAttn, dorsal attention network; SalVentAttn, salience/ventral attention network; Limbic, limbic network; Cont, frontoparietal control network; Default, default mode network; Sub, subcortical network

© 2025. Cao R et al. Published online at http://www.aginganddisease.org/EN/10.14336/AD.2025.0428

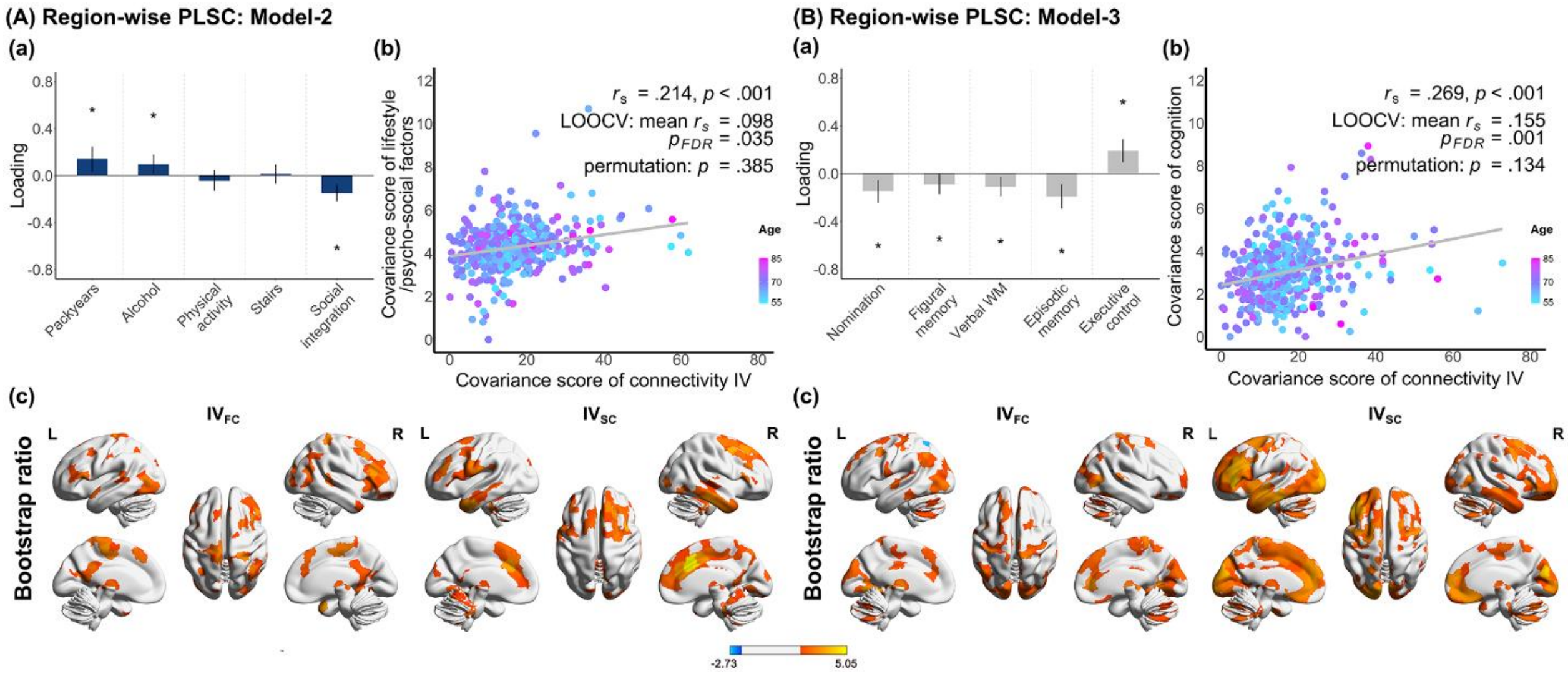

Supplementary Figure 3. Model 2 (A) and Model 3 (B) of region-wise PLSC analysis in the whole group. The bar plots show the loading of lifestyle/psycho-social factors [A (a)] or cognition [B (a)], with 95% confidence intervals calculated via bootstrap resampling. Significant contributors are marked with an asterisk (*). A scatter plot shows the correlation between covariance scores of individual variability (IV) of connectivity (*X*-axis) and covariance scores (*Y*-axis) of lifestyle/psycho-social factors [A (b)] or cognition [B (b)], with participant ages color-coded (55 to 85 years old, light blue to pink). The bootstrap ratio brain maps [A (c) and B (c)] represent region-wise connectivity IV covariance patterns. Positive (in red-yellow) or negative (in blue) bootstrap ratios indicate regions' positive or negative contributions to the overall pattern. Only significantly contributed regions are shown (bootstrap ratio >1.96 or < -1.96). Confounders (age, sex, education) were regressed out before PLSC. Abbreviations: r_s, Spearman correlation coefficient; $IV_{FC/SC}$, individual variability of functional/ structural connectivity; Packyears, packyears of smoking; Alcohol, alcohol consumption; Physical activity, measured by metabolic equivalent of the task; Stairs, flights of stairs climbed; WM, working memory

© 2025. Cao R et al. Published online at http://www.aginganddisease.org/EN/10.14336/AD.2025.0428

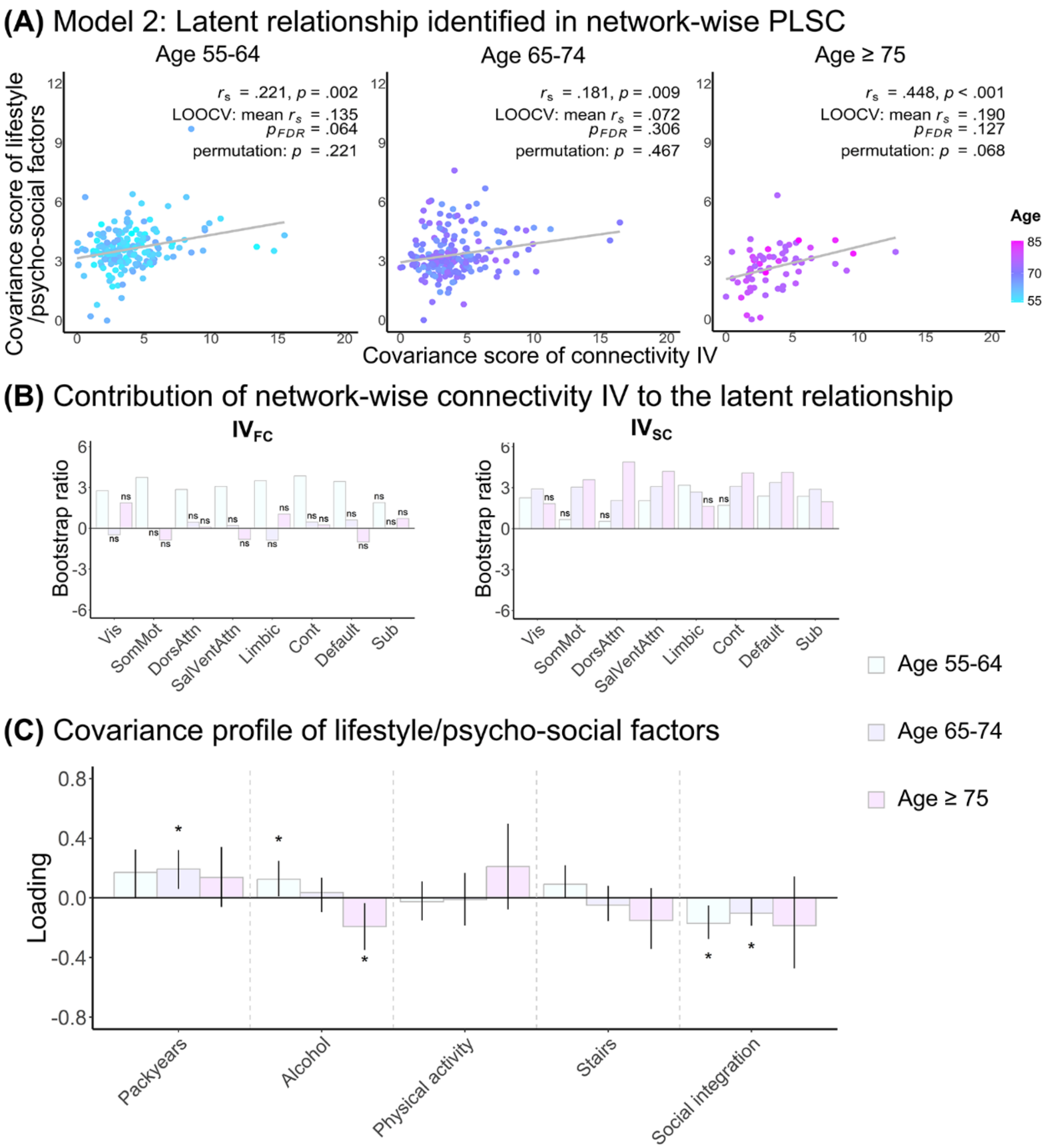

Supplementary Figure 4. Model 2 of network-wise PLSC analysis across three age subgroups. (A) Scatter plot correlating covariance scores of individual variability (IV) of connectivity (*X*-axis) with covariance scores of lifestyle/psycho-social factors (*Y*-axis) for the age groups 55-64 (light blue), 65-74 years (purple), and ≥ 75 years (pink), with darker shades representing older ages in the respective age group. (B) Bar charts of bootstrap ratio indicating IV of functional (IV_{FC}, left) and structural connectivity (IV_{SC}, right). Positive/negative ratios signify positive/negative contributions to the overall pattern. 'ns' indicates non-significant ratios (-1.96 < ratio < 1.96). (C) Loadings of lifestyle/psycho-social variables with 95% bootstrap confidence intervals. Significant contributors are marked with *. Colors match (A); faded bars indicate results not confirmed in validation analysis (e.g., age 55-64). Confounders (age, sex, education) were regressed out before PLSC. Abbreviations: r_s, Spearman correlation coefficient; Packyears, packyears of smoking; Alcohol, alcohol consumption; Physical activity, measured by metabolic equivalent of the task; Stairs, flights of stairs climbed; Vis, visual network; SomMot, somatomotor network; DorsAttn, dorsal attention network; SalVentAttn, salience/ventral attention network; Limbic, limbic network; Cont, frontoparietal control network; Default, default mode network; Sub, subcortical network

© 2025. Cao R et al. Published online at http://www.aginganddisease.org/EN/10.14336/AD.2025.0428

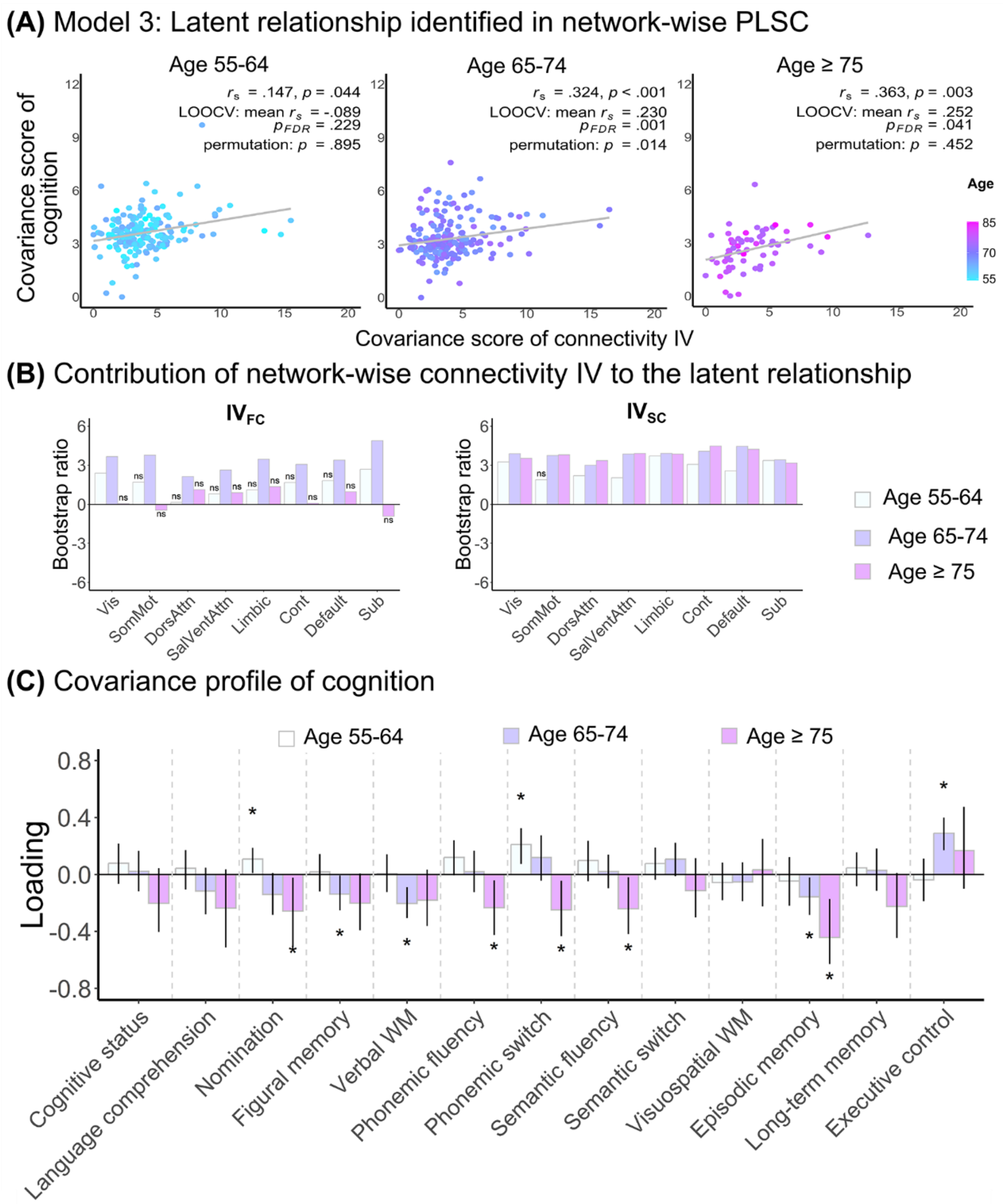

Supplementary Figure 5. Model 3 of network-wise PLSC analysis across three age subgroups. (A) Scatter plot correlating covariance scores of individual variability (IV) of connectivity (*X*-axis) and covariance scores of cognition (*Y*-axis) for the age groups 55-64 years (light blue), 65-74 years (purple), and ≥ 75 years (pink), with darker shades representing older ages in the respective age group. (B) Bar charts of bootstrap ratio indicating IV of functional (IV_{FC}, left) and structural connectivity (IV_{SC}, right). Positive/negative ratios signify positive/negative contributions to the overall pattern. 'ns' indicates non-significant ratios (-1.96 < ratio < 1.96). (C) Loadings of cognitive variables with 95% bootstrap confidence intervals. Significant contributors are marked with *. Colors match (A); faded bars indicate results not confirmed in validation analysis (e.g., age 55-64). Confounders (age, sex, education) were regressed out before PLSC. Abbreviations: r_s, Spearman correlation coefficient; WM, working memory; Vis, visual network; SomMot, somatomotor network; DorsAttn, dorsal attention network; SalVentAttn, salience/ventral attention network; Limbic, limbic network; Cont, frontoparietal control network; Default, default mode network; Sub, subcortical network

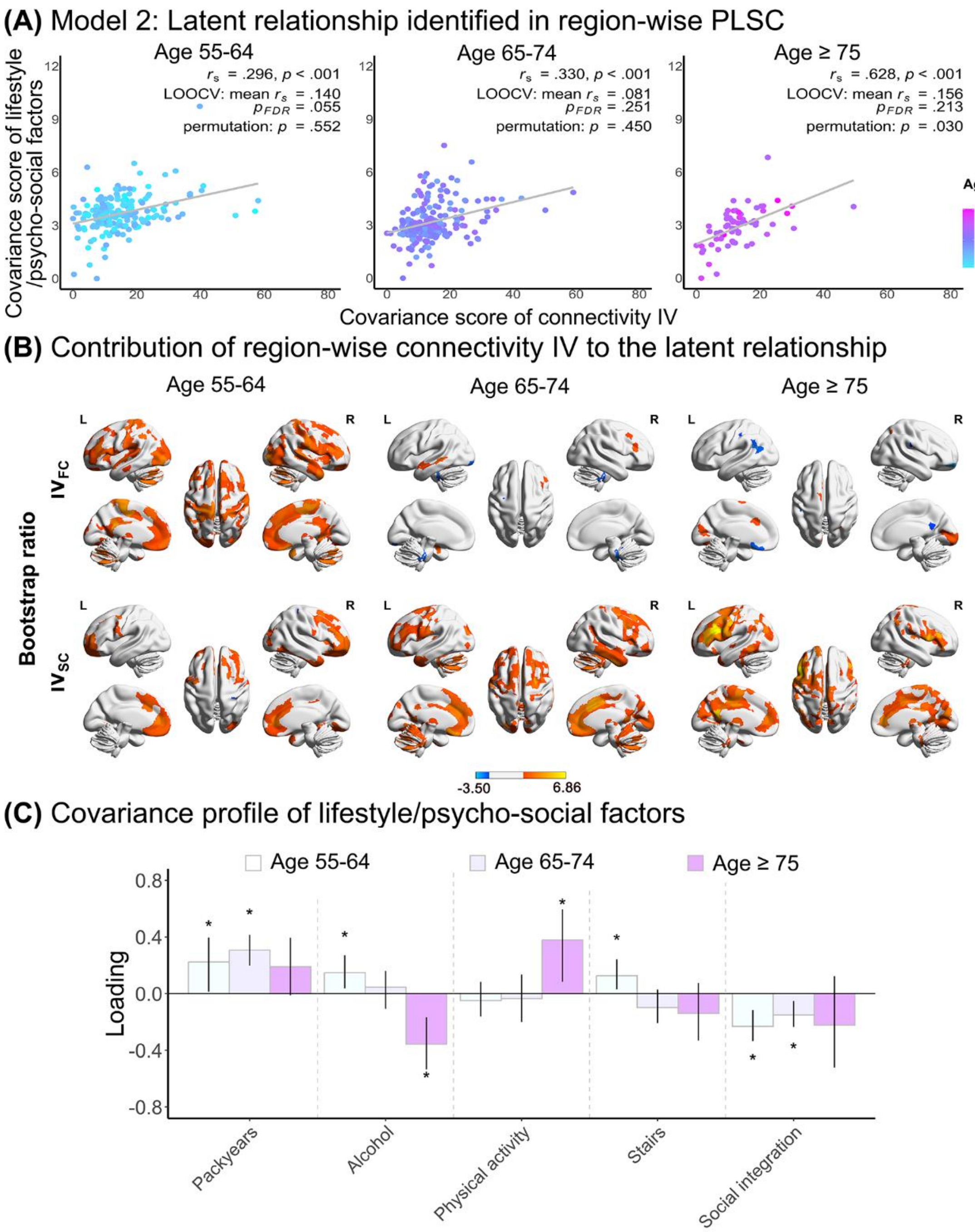

Supplementary Figure 6. Model 2 of region-wise PLSC analysis across three age subgroups. (A) Scatter plot correlating covariance scores of individual variability (IV) of connectivity (*X*-axis) and covariance scores of lifestyle/psycho-social factors (*Y*-axis) for the age groups 55-64 years (light blue), 65-74 years (purple), and ≥ 75 years (pink), with darker shades representing older ages in the respective age group. (B) Bootstrap ratio brain maps representing region-wise IV_{FC} (upper) and IV_{SC} (lower) covariance patterns for three age groups. Positive (in red-yellow) or negative (in blue) ratios indicate brain regions' positive or negative contributions to the overall pattern. Only significantly contributed regions are displayed (bootstrap ratio >1.96 or < -1.96). (C) Loadings of lifestyle/psycho-social variables with 95% bootstrap confidence intervals. Significant contributors are marked with an asterisk (*). Colors match (A); faded bars indicate results not confirmed in validation analysis (e.g., age 55-64). Confounders (age, sex, education) were regressed out before PLSC. Abbreviations: $IV_{FC/SC}$, individual variability of functional/ structural connectivity; r_s, Spearman correlation coefficient; Packyears, packyears of smoking; Alcohol, alcohol consumption; Physical activity, measured by metabolic equivalent of the task; Stairs, flights of stairs climbed

© 2025. Cao R et al. Published online at http://www.aginganddisease.org/EN/10.14336/AD.2025.0428

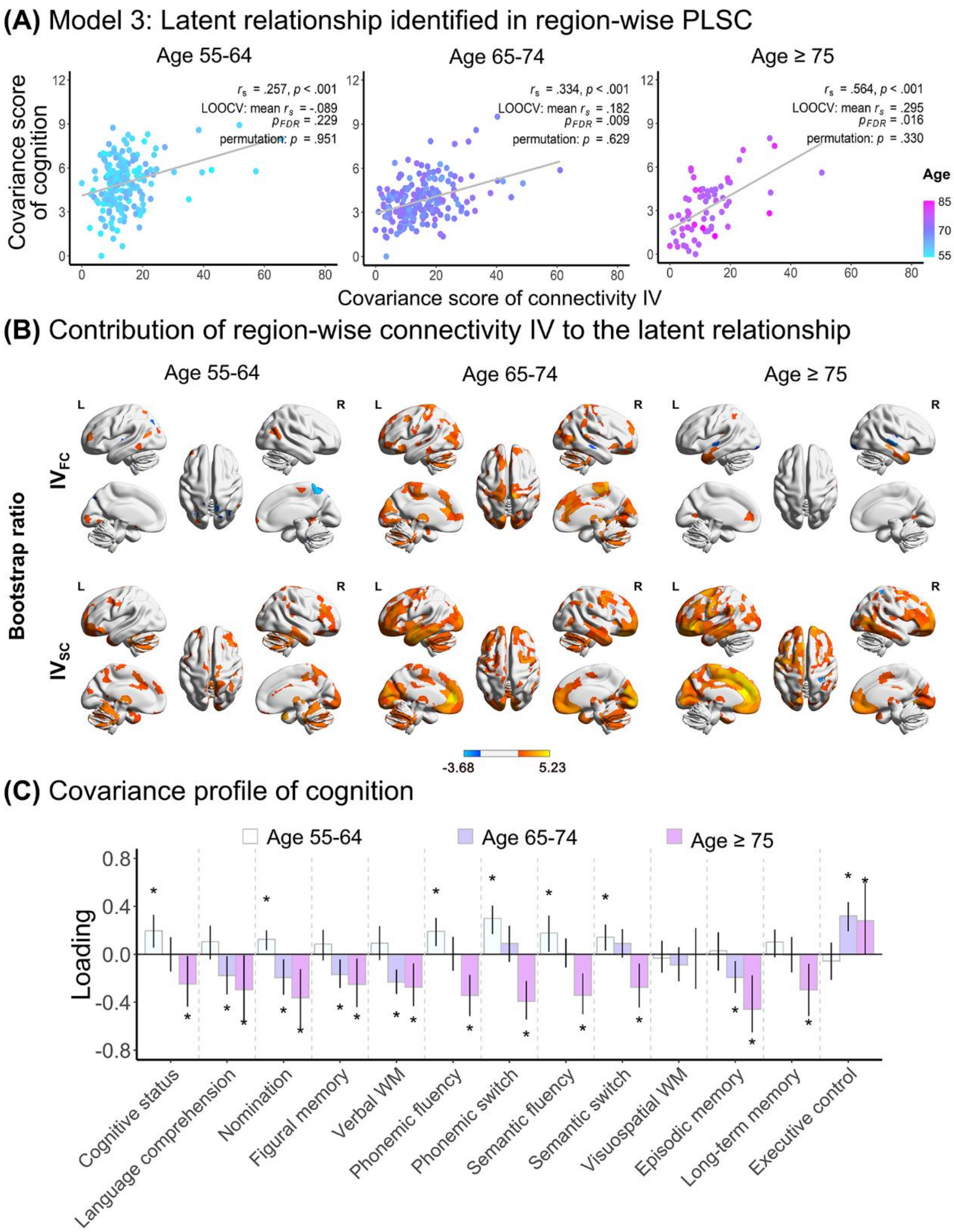

Supplementary Figure 7. Model 3 of region-wise PLSC analysis across three age subgroups. (A) Scatter plot correlating covariance scores of individual variability (IV) of connectivity (*X*-axis) and covariance scores of cognition (*Y*-axis) for the age groups 55-64 years (light blue), 65-74 years (purple), and ≥ 75 years (pink), with darker shades representing older ages in the respective age group. (B) Bootstrap ratio brain maps representing region-wise IV_{FC} (upper) and IV_{SC} (lower) covariance patterns for three age groups. Positive (in red-yellow) or negative (in blue) ratios indicate brain regions' positive or negative contributions to the overall pattern. Only significantly contributed regions are displayed (bootstrap ratio >1.96 or < -1.96). (C) Loadings of cognitive variables with 95% bootstrap confidence intervals. Significant contributors are marked with *. Colors match (A); faded bars indicate results not confirmed in validation analysis (e.g., age 55-64). Confounders (age, sex, education) were regressed out before PLSC. Abbreviations: $IV_{FC/SC}$, individual variability of functional/ structural connectivity; r_s, Spearman correlation coefficient; WM, working memory

© 2025. Cao R et al. Published online at http://www.aginganddisease.org/EN/10.14336/AD.2025.0428

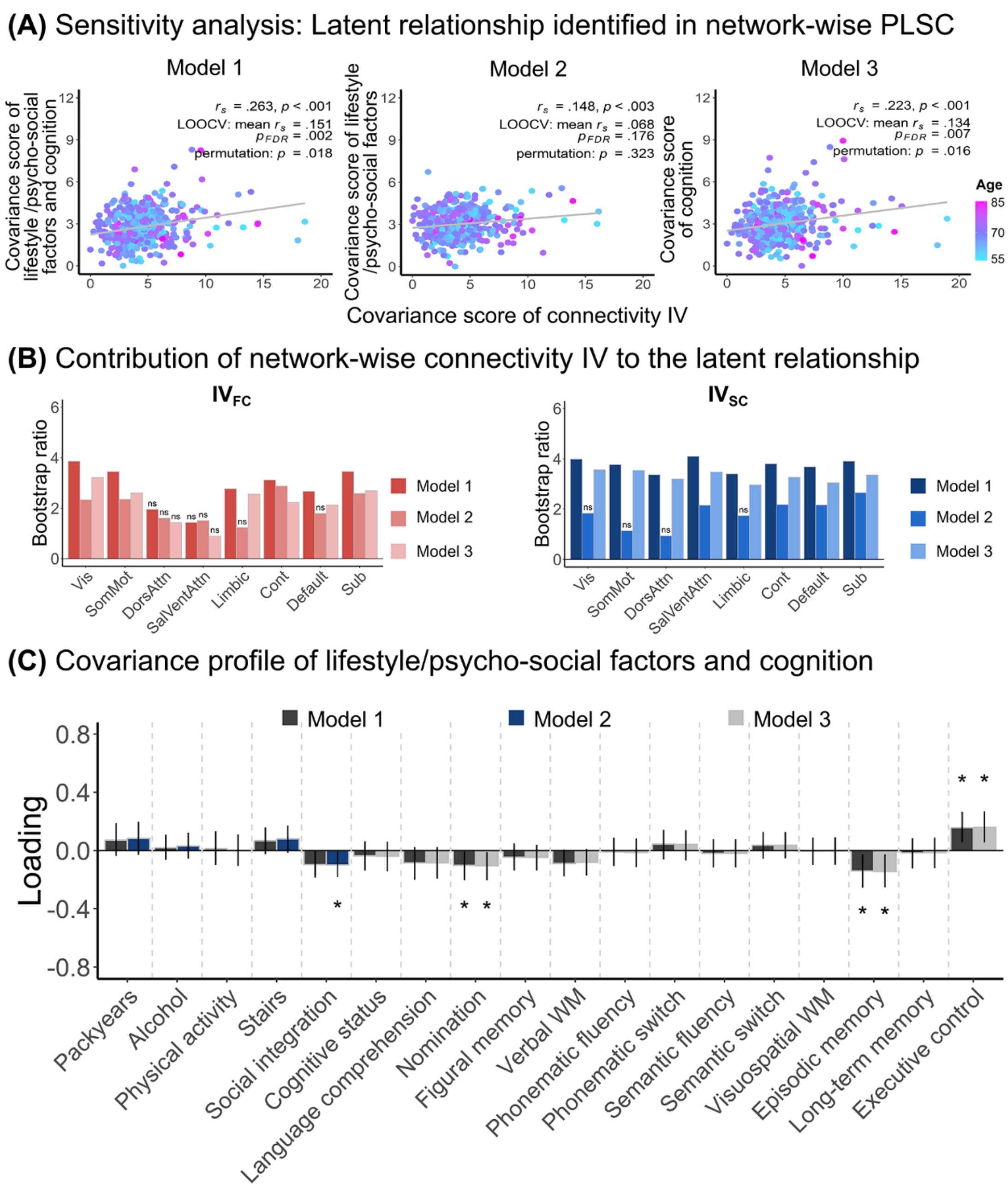

Supplementary Figure 8. Sensitivity analysis: network-wise PLSC analysis in 403 participants. (A) Scatter plot illustrating the covariance scores of individual variability (IV) of connectivity (*X*-axis) and covariance scores of lifestyle/psycho-social factors and/or cognition (*Y*-axis) for the three models, with participant ages color-coded from light blue to pink. (B) Bar charts of the bootstrap ratio depicting IV of functional (IV_{FC}, left) and structural connectivity (IV_{SC}, right). Positive/negative ratios indicate contributions to the overall covariance pattern. 'ns' denotes non-significant ratios (-1.96 < ratio < 1.96). (C) Loadings of lifestyle/psycho-social and/or cognitive variables with 95% bootstrap confidence intervals. Significant contributors are marked with an asterisk (*). Black represents Model 1, blue represents Model 2, and gray represents Model 3. Confounders (age, sex, education) were regressed out before PLSC. The results in Model 2 were not confirmed in the validation analysis. Abbreviations: r_s, Spearman correlation coefficient; Packyears, packyears of smoking; Alcohol, alcohol consumption; Physical activity, measured by metabolic equivalent of the task; Stairs, flights of stairs climbed; WM, working memory; Vis, visual network; SomMot, somatomotor network; DorsAttn, dorsal attention network; SalVentAttn, salience/ventral attention network; Limbic, limbic network; Cont, frontoparietal control network; Default, default mode network; Sub, subcortical network

© 2025. Cao R et al. Published online at http://www.aginganddisease.org/EN/10.14336/AD.2025.0428

SUPPLEMENTARY DATA

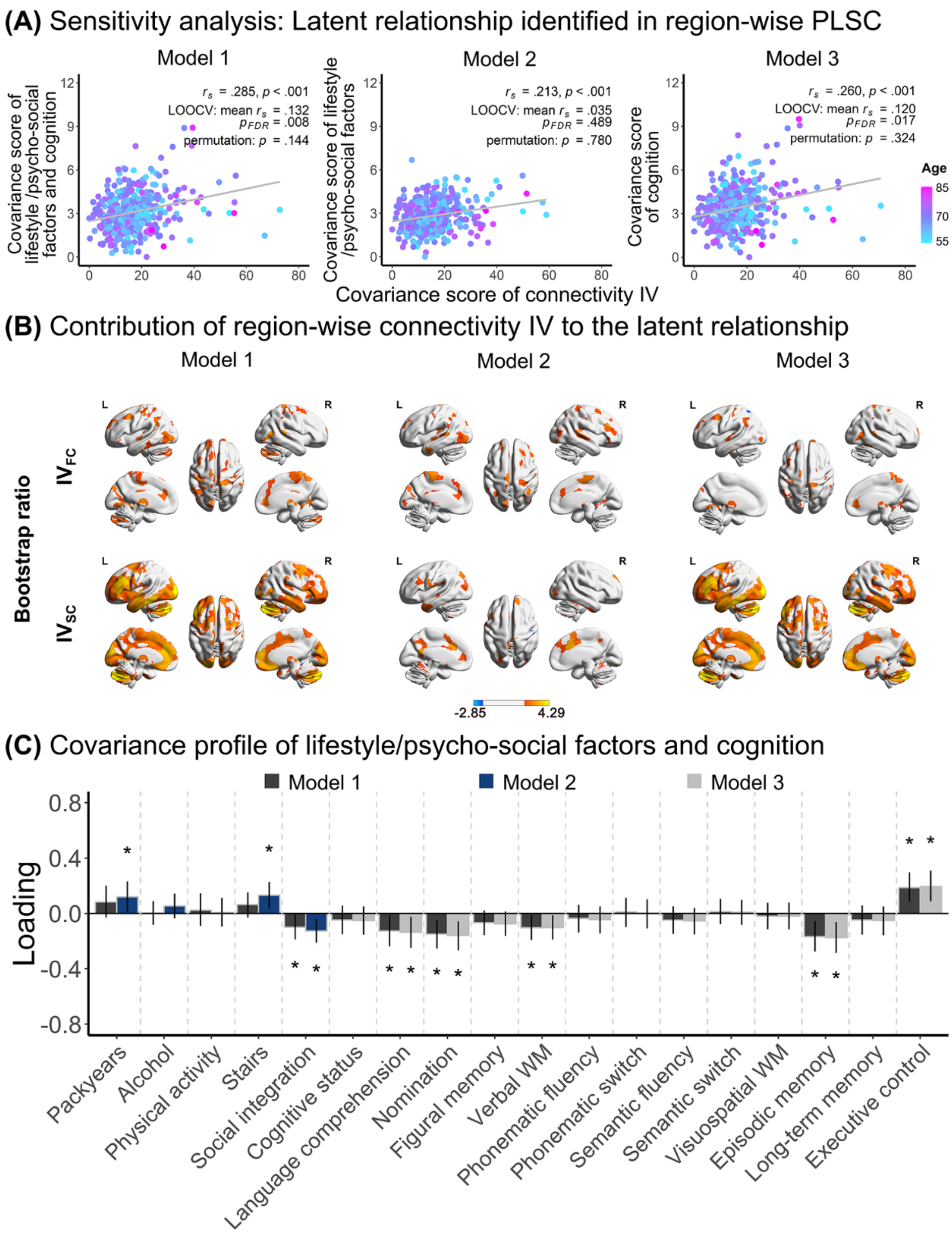

Supplementary Figure 9. Sensitivity analysis: region-wise PLSC analysis in 403 participants. (A) Scatter plot depicting the covariance scores of individual variability (IV) of connectivity (*X*-axis) and covariance scores of lifestyle/psycho-social factors and/or cognition (*Y*-axis) for the three models, with participant ages color-coded from light blue to pink. (B) Bar charts of the bootstrap ratio illustrating IV of functional (IV_{FC}, upper) and structural connectivity (IV_{SC}, lower). Positive/negative ratios indicate contributions to the overall covariance pattern. Only significantly contributed regions are displayed (bootstrap ratio >1.96 or < -1.96). (C) Loadings of lifestyle/psycho-social and/or cognitive variables with 95% bootstrap confidence intervals. Significant contributors are marked with an asterisk (*). Black represents Model 1, blue represents Model 2, and gray represents Model 3. Confounders (age, sex, education) were regressed out before PLSC. The results in Model 2 were not confirmed in the validation analysis. Abbreviations: r_s, Spearman correlation coefficient; Packyears, packyears of smoking; Alcohol, alcohol consumption; Physical activity, measured by metabolic equivalent of the task; Stairs, flights of stairs climbed; WM, working memory

© 2025. Zhang M et al. Published online at http://www.aginganddisease.org/EN/10.14336/AD.2025.0428

SUPPLEMENTARY DATA

eReferences

[1] Stumme J, Kramer C, Miller T, Schreiber J, Caspers S, Jockwitz C (2022). Interrelating differences in structural and functional connectivity in the older adult's brain. Hum Brain Mapp, 43:5543–5561.
[2] Pruim RHR, Mennes M, van Rooij D, Llera A, Buitelaar JK, Beckmann CF (2015). ICA-AROMA: A robust ICA-based strategy for removing motion artifacts from fMRI data. Neuroimage, 112:267–277.
[3] Ashburner J, Friston KJ (2005). Unified segmentation. Neuroimage, 26:839–851.
[4] Afyouni S, Nichols TE (2018). Insight and inference for DVARS. Neuroimage, 172:291–312.
[5] Stumme J, Jockwitz C, Hoffstaedter F, Amunts K, Caspers S (2020). Functional network reorganization in older adults: Graph-theoretical analyses of age, cognition and sex. Neuroimage, 214:116756.
[6] Gaser C, Dahnke R, Thompson PM, Kurth F, Luders E, The Alzheimer's Disease Neuroimaging I (2024). CAT: a computational anatomy toolbox for the analysis of structural MRI data. Gigascience, 13:giae049.
[7] Mueller S, Wang D, Fox MD, Yeo BT, Sepulcre J, Sabuncu MR*, et al.* (2013). Individual variability in functional connectivity architecture of the human brain. Neuron, 77:586–595.
[8] Sun L, Liang X, Duan D, Liu J, Chen Y, Wang X*, et al.* (2022). Structural insight into the individual variability architecture of the functional brain connectome. Neuroimage, 259:119387.
[9] DuPre E, Spreng RN (2017). Structural covariance networks across the life span, from 6 to 94 years of age. Netw Neurosci, 1:302–323.
[10] Petersen M, Hoffstaedter F, Nagele FL, Mayer C, Schell M, Rimmele DL*, et al.* (2024). A latent clinical-anatomical dimension relating metabolic syndrome to brain structure and cognition. Elife, 12:RP93246.
[11] Kalbe E, Kessler J, Calabrese P, Smith R, Passmore AP, Brand M*, et al.* (2004). DemTect: a new, sensitive cognitive screening test to support the diagnosis of mild cognitive impairment and early dementia. Int J Geriatr Psych, 19:136–143.
[12] Schmidt KH, Metzler P (1992). Wst-Wortschatztest. Göttingen: Beltz Test, 16.
[13] Morris JC, Heyman A, Mohs RC, Hughes JP, van Belle G, Fillenbaum G*, et al.* (1989). The Consortium to Establish a Registry for Alzheimer's Disease (CERAD). Part I. Clinical and neuropsychological assessment of Alzheimer's disease. Neurology, 39:1159–1165.
[14] Benton AL, Benton-Sivan A, Spreen O, Streck P (2009). Der Benton-Test. Göttingen: Verlag Hans Huber.
[15] Oswald WD, Fleischmann UM (1997). Nürnberger-Alters-Inventar (NAI). Göttingen: Hogrefe.
[16] Aschenbrenner S, Tucha O, Lange KW (2000). Der Regensburger Wortflüssigkeits-Test (RWT). Göttingen: Hogrefe.
[17] Schelling D (1997). Block-Tapping-Test. Frankfurt: Swets Test Services.
[18] Lux S, Hartje W, Reich C, Nagel C (2012). VGT: Verbaler Gedächtnistest: Bielefelder Kategorielle Wortlisten. Göttingen: Verlag Hans Huber.
[19] Caspers S, Moebus S, Lux S, Pundt N, Schutz H, Muhleisen TW*, et al.* (2014). Studying variability in human brain aging in a population-based German cohort-rationale and design of 1000BRAINS. Front Aging Neurosci, 6:149.

© 2025. Zhang M et al. Published online at http://www.aginganddisease.org/EN/10.14336/AD.2025.0428

SUPPLEMENTARY DATA

© 2025. Zhang M et al. Published online at http://www.aginganddisease.org/EN/10.14336/AD.2025.0428
